# Enhanced ion intercalation in Ni_*x*_K_1−2*x*_TiNbO_5_ enabled by redox active Ni exchange for potassium-ion batteries

**DOI:** 10.1039/d5sc04984a

**Published:** 2025-09-15

**Authors:** Charlie A. F. Nason, Ajay Piriya Vijaya Kumar Saroja, Wanjun Ren, Yingkangzi Mei, Asma Sarguroh, Yupei Han, Yi Lu, Jamie A. Gould, Tim I. Hyde, Veronica Celorrio, Gopinathan Sankar, Yang Xu

**Affiliations:** a Department of Chemistry, University College London 20 Gordon Street London WC1H 0AJ UK y.xu.1@ucl.ac.uk; b Department of Chemistry, The University of Southampton SO9 5NH UK; c Diamond Light Source, Harwell Science and Innovation Campus Didcot OX11 0DE UK

## Abstract

The ultimate goal of potassium-ion batteries (KIBs) is to become a serious competitor to lithium-ion batteries (LIBs). Achieving this requires the development of high energy density negative electrode materials, with transition metal oxides emerging as the most promising candidates. However, despite their high theoretical capacities, most transition metal oxides still struggle to achieve high performance, often necessitating substantial nanostructuring. Ion-exchange presents a facile and effective process for enhancing material properties, yet the demonstration of the exchanged ions undergoing redox activity has not been previously reported for KIBs. Herein, this work reports Ni_0.25_K_0.5_TiNbO_5_, synthesized through the ion-exchange between K^+^ and Ni^2+^, as a novel negative electrode material for KIBs. The ion-exchanged material achieves a specific capacity of 304 mAh g^−1^ in the first cycle and 162 mAh g^−1^ after 10 cycles, corresponding to a 240% and 156% increase compared to the pristine, unexchanged KTiNbO_5_ at the same cycle numbers. The structure–performance relationship was investigated in detail, shedding light on the previously unknown relationships between the level of hydration, degree of exchange and the performance of ion-exchanged materials. Furthermore, the exchanged Ni was demonstrated to be reversibly redox active, contributing to the observed capacity and representing a first for ion-exchanged materials in the KIB literature.

## Abstract 2

The ion-exchange of structural K^+^ in KTiNbO_5_ with Ni^2+^ can increase the electrochemical intercalation of K^+^, enhancing the performance as a negative electrode material for potassium-ion batteries.

## Graphical abstract

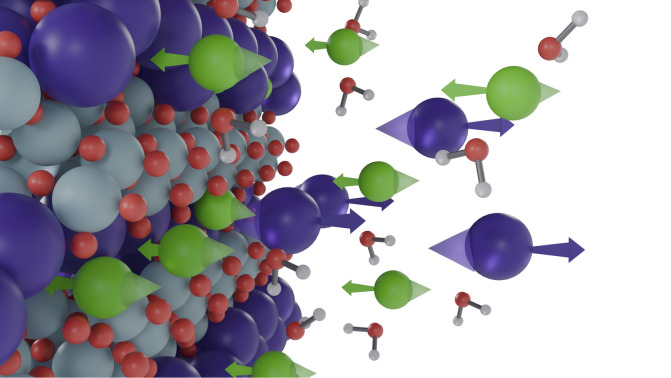

## Introduction

Potassium-ion batteries (KIBs) are rapidly developing as a promising alternative to both lithium-ion (LIBs) and sodium-ion batteries (NIBs).^1^ There has been a concerted effort in the academic community to improve electrode materials that determine many of the properties of the final battery, with the goal of producing a high energy density, stably cycling and high specific capacity electrode material. This goal has numerous environmental, cost and energy security benefits, which have been demonstrated at length in the literature, but mostly stems from the crustal abundance and global distribution of potassium *vs.* lithium (20 900 ppm *vs.* 20 ppm).^2^ However, despite the advantages that KIBs offer, significant shortcomings still hinder widespread commercialization. Many of these issues stem from the size and mass of K^+^ (1.33 Å, 39.098a.u.), which result in sluggish diffusion in solids and substantial volume changes in the host lattice.^3–6^ These limitations reduce the energy density, rate capability and cycling stability of electrode materials, all of which are essential factors in the performance of an energy storage system.

Transition metal oxides (TMOs) have been extensively investigated to address these problems, as certain transition metal compounds have large interlayer spacings and channels, allowing for fast diffusion and reduced expansion and contraction on (de)intercalation, despite the large size of K^+^.^7–9^ Combined with the redox activity of the transition metal ions providing large theoretical capacities, TMOs are a proven family of electrode materials, with materials such as lithium titanates (Li_4_Ti_5_O_12_, LTO), nickel manganese cobalt oxides (LiNi_*x*_Mn_*y*_Co_1−*x*−*y*_O_2_, NMC) and sodium titanates (Na_2_Ti_3_O_7_, NTO) having widespread commercial and research success in previous years.^10,11^ For the negative electrode in KIBs, most TMOs in the literature are based on niobium or titanium oxides, as their common redox potentials fall in the anodic range, 0–1 V (*vs.* K^+^/K), maximizing energy density of the overall cell. However, the high cost of transition metals like niobium, combined with the limited electrochemical performance of TMO negative electrodes, currently makes them uncompetitive with graphite, a material that is inexpensive, well understood and exhibits excellent electrochemical performance at low rates (280 mAh g^−1^).^4^ Improving both the capacity and the rate performance of TMOs is of paramount importance. One pathway to further develop these materials is through the process of ion-exchange.

Ion-exchange involves the reversible substitution of one ion in one medium with an alternative ion of the same sign charge from a second medium, as represented in eqn (1), with the most common form being an insoluble solid placed in an aqueous salt solution.^12,13^

1 *mA*_(s)_^*n*+^ + *nB*_(aq)_^*m*+^ ⇌ *nB*_(s)_^*m*+^ + *mA*_(aq)_^*n*+^

This process is driven by electrostatic interactions and governed by thermodynamic equilibrium, where the selectivity for specific ions is described by selectivity coefficients and is influenced by ion size, charge, hydration energy, active site coordination energy and sterics.^13^ While thermodynamics governs the distribution of ions between the two media, kinetics dictates the rate of ion-exchange and when performed with an insoluble material, is often limited by the solid-state diffusion in the material. This ion-exchange process is widely applied in resins for water softening, nuclear waste treatment and metal recovery, growing into a multi-billion dollar industry, but is yet to be applied at a wide scale in the energy storage industry.^14–16^

The research on the use of ion-exchange as an approach to improving battery materials has begun to accelerate in recent years, but is still relatively underdeveloped.^17–19^ This is in part because it requires several inherent prerequisites; the material must have ions to exchange, be able to de-intercalate these ions while maintaining its structural integrity and finally be stable under aqueous conditions, as many ion-exchanges are performed in water. This explains the focus of work on ion-exchange for battery materials, as these conditions exclude many common negative and positive electrode materials. There is a much larger body of work focusing on cathode materials such as NMC and vanadium oxides, with negative electrode materials rarely researched.^17^ Furthermore, the vast majority of the ion-exchange works focus on switching a material to an alternative battery chemistry, for example, exchanging K^+^ in a material with Na^+^ for use in sodium-ion batteries.^19^ A novel approach is to exchange a material with a redox active ion, which provides additional redox activity to the electrochemical performance of the material. To the best of our knowledge, this approach has not been covered in the ion-exchange electrochemical energy storage literature previously.

Previously, our group has reported a solvothermally synthesized KTiNbO_5_ (KTNO)/reduced graphene oxide (rGO) nanocomposite as an intercalation-type TMO negative electrode material for KIBs and demonstrated that K^+^ can be reversibly intercalated and deintercalated, accompanied Ti and Nb redox activity.^20^ However, KTNO still faces challenges as a potential high-performance negative electrode material, as it suffers from low capacity relative to its theoretical capacity (206 mAh g^−1^ based on 2 e^−^ transfer) and sluggish kinetics. KTNO is ideally suited for ion-exchange, as it fulfils all the criteria. It is moisture stable and has been previously researched as an ion-exchange material, in the energy storage and other areas, with H^+^, Li^+^, Mn^2+^, Ni^2+^, Sr^3+^, Al^3+^, Pb^2+^, Ba^2+^, Sb^3+^ and Mg^2+^ being shown to be exchanged.^18,21–28^ of these, H^+^, Li^+^, Al^3+^, Pb^2+^, Ba^2+^, Sb^3+^ and Mg^2+^ have been electrochemically tested in both LIBs and NIBs. Thus, KTNO can accommodate a variety of different ions in its interlayer space and by leveraging this property, the structure–performance relationship can be tuned to enhance its electrochemical performance. The crystal structure of KTNO is the key to this ion-exchange ability, as it crystallizes in an orthorhombic crystal system with a layered structure made up of corrugated 2×3×^∞^ slabs of TiO_6_ octahedra and NbO_6_ octahedra, forming channels that are filled with K^+^.^29^ The K^+^, coordinated in octahedral sites, is highly mobile and can be readily exchanged. By exchanging large K^+^ ions with smaller, redox active multivalent ions, the electrochemical performance could be significantly enhanced.

Herein, through a facile and highly controllable ion-exchange synthesis, a tunable product Ni_*x*_K_1−*x*_TiNbO_5_ (Ni-KTNO), was synthesized. The structure, along with the electrochemical performance and charge storage mechanism of Ni-KTNO with various nickel contents, was investigated and demonstrated enhanced electrochemical performance. It was determined that the enhancement originated from an increase in the interlayer spacing due to the presence of co-intercalated water molecules, along with the activation of the newly available Ni^2+^/Ni^0^ redox couple. The specific capacity of Ni_0.25_K_0.5_TiNbO_5_ increased by 240%, exhibiting 304 mAh g^−1^ at 10 mA g^−1^ on the first charge, compared to the pristine KTNO (127 mAh g^−1^). X-ray absorption spectroscopy (XAS) analysis showed that the ion-exchanged nickel ions are redox active, being reversibly reduced to Ni^0^ and re-oxidized to Ni^2+^.

## Experimental

### Preparation of KTNO and Ni-KTNO

KTNO was prepared *via* solid-state synthesis. Titanium dioxide (TiO_2_, 3.01 g, 99.9%, Sigma Aldrich), niobium oxide (Nb_2_O_5_, 5.00 g, 99.8%, Sigma Aldrich) and potassium carbonate (K_2_CO_3_, 2.73 g, 99%, Sigma Aldrich) and 10 ml of isopropyl alcohol (IPA) was added to a ZrO_2_ lined ball milling jar (80 ml), along with 1 cm diameter balls and ball milled together to form a homogeneous slurry (700 rpm, 5 min running/2 min rest x2, Pulverisette 7, Fritsch). This slurry was then dried and calcinated in air at 500 °C for 2 h, then 1000 °C for a further 8 h (5 °C min^−1^ ramp rate). The resulting white powder was then ball milled (500 rpm, 5 min running/2 min rest x2, 1 : 1 volume ratio with IPA) to reduce the particle size and break up hard aggregates. In a typical ion-exchange process, KTNO powder was added to 0.25 M solutions of nickel chloride (NiCl_2_, 98%, Sigma Aldrich) and stirred for 5 h at 80 °C at various ratios of K : Ni, after which the samples were centrifuged at 6000 rpm for 5 min and washed 3× with deionized water. The resulting powder was dried at 120 °C overnight or 60 °C for 1 h or 400 °C for 5 h, depending on the level of hydration.

### Materials characterization

Powder X-ray diffraction (XRD) and in-*operando* XRD were carried out on a Stoe STADI-P diffractometer using Mo Kα1 radiation (0.7093 Å, 50 kV, 25 mA) at a scan rate of 30 s degree, between 2.000° and 40.115° or 32.185° or Cu Kα1 radiation (1.54060 Å, 40 kV, 30 mA) at a scan rate of 30 s degree, between 2.000° and 80.195°, then converted to Mo wavelength. Rietveld refinements were carried out using the GSAS-II software package. Thermal gravimetric analysis (TGA) was performed on a PerkinElmer Simultaneous Thermal Analyzer (STA) 6000 (20 ml min^−1^, 800 °C, 10 °C min^−1^). DLS was performed on a Malvern Zetasizer. Scanning electron microscopy (SEM) and energy-dispersive X-ray spectroscopy (EDS) analysis were performed on a Jeol JSM 7600 Field Emission Gun – Scanning Electron Microscope. Transmission electron microscopy (TEM) analysis was performed on a Jeol 2100 transmission electron microscope. X-ray photoelectron spectra (XPS) were recorded on a ThermoScientific Kα X-ray Photoelectron Spectrometer, with charging correction applied using C 1s as a reference at 285.00 eV. Etching was performed at 10 s per level for 9 levels, at an ion energy of 3 keV. X-ray absorption spectroscopy (XAS) at the Ni, Nb and Ti K-edges were recorded at beamline B18 at Diamond Light Source (UK). The electrode samples were sealed in Kapton tape under glovebox conditions. The Ni, Nb and Ti K-edges were measured using the Si (111) crystal monochromator, switching from a Pt mirror coating for the Ni and Ti edges to a Cr mirror coating for the Nb edge. The pristine samples and reference samples were pelletized before the measurements. The spectra were collected in fluorescence and transmission mode. The obtained X-ray absorption near-edge spectra (XANES) and extended X-ray absorption fine structure (EXAFS) data were processed using the Demeter software package.

### Electrochemical measurements

Electrode slurries were prepared in a 70 : 20 : 10 wt% ratio of active material : super P : sodium carboxymethylcellulose (Na-CMC), which were then coated *via* the doctor blade technique onto carbon-coated aluminium foil, then dried under vacuum at 60 °C overnight. Due to these drying conditions, phase II was used for main electrochemical tests. Mass loadings of active materials on 16 mm diameter electrode disks were between 1–2 mg cm^−2^ for all electrochemical analysis. CR2032 stainless steel coin cells were assembled in an Ar filled glovebox (O_2_ and H_2_O < 0.1 ppm) with K or Na metal as the counter electrode, glass microfiber (Whatman, Grade F) as the separator and 2.5 M potassium bis(fluorosulfonyl)imide (KFSI) or sodium bis(fluorosulfonyl)imide (NaFSI) in triethylphosphate (TEP) as the electrolyte. Cyclic voltammetry (CV) and electrochemical impedance spectroscopy (EIS) were measured on a Biologic VSP potentiostat (0.01–3 V *vs.* K/K^+^). Galvanostatic charge–discharge (GCD) profiles were measured on Neware battery cyclers (0.01–3 V *vs.* K/K^+^ or Na/Na^+^). The in-*operando* cells were prepared using an AMPIX cell, with a glassy carbon window. A sample displacement correction was applied to account for the added distance between the sample and the detector.^30^ Galvanostatic intermittent titration technique (GITT) analysis was performed using Neware cyclers, with 20-minute pulses of 10 mA g^−1^, followed by a rest of 2 h.

## Results and discussions

### Material synthesis and characterization

**Fig. 1:**
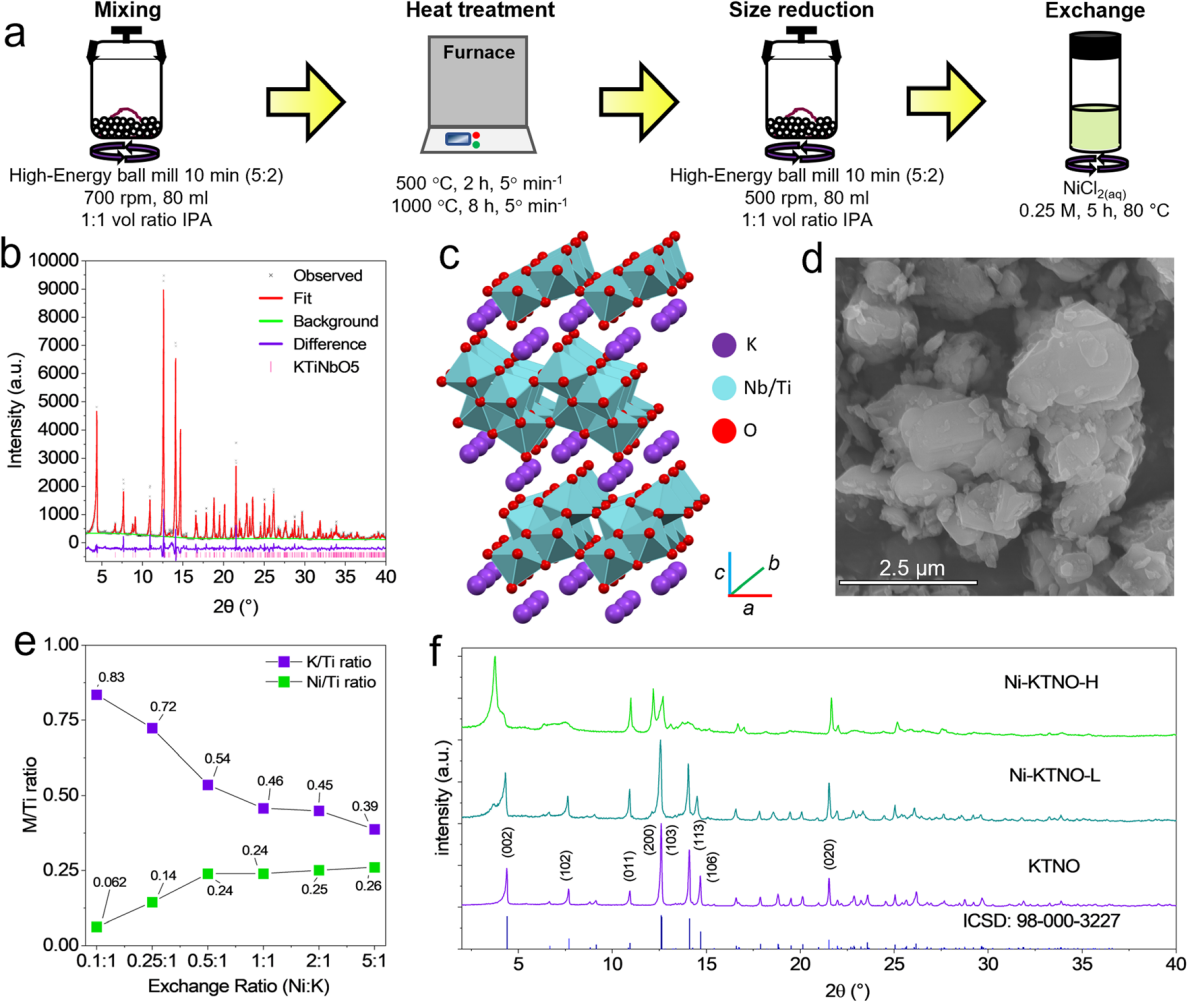
(a) Synthesis schematic. (b) Rietveld refinement fit to XRD pattern of KTNO. (c) Crystal structure schematic of KTNO. (d) SEM image of KTNO. (e) EDS ratios of Ni-KTNO synthesized at exchange ratios from 0.1 : 1 to 5 : 5 Ni/K. (f) XRD patterns of KTNO, Ni-KTNO-L and Ni-KTNO-H.

*Via* the process shown in Fig. 1a, the pristine KTNO and the ion-exchanged Ni-KTNO were synthesized. This involves wet-milling TiO_2_, Nb_2_O_5_ and K_2_CO_3_ in a 1 : 1 : 1.05 ratio at 700 rpm (5 min on:2 min rest x2) to homogenize the precursors, then calcinating for 2 h at 500 °C, then 8 h at 1000 °C. This was then milled at 500 rpm (5 min on:2 min rest x2) to break up hard aggregates that had formed in the calcination process. This second milling step had the additional benefit of reducing the particle size to improve the kinetics of the electrochemical reaction, as the smaller particles have a shorter diffusion path length for K^+^ intercalation. Fig. 1b shows the XRD pattern of the post-milling KTNO. The pattern shows high crystallinity and high phase purity, well matching the standard KTNO pattern (ICSD: 98-000-3227). A Rietveld refinement was carried out, with the obtained lattice parameters summarized in Table S1, with an *R*_wp_ of 10.75%, with a schematic of the crystal structure shown in Fig. 1c. The milling process was performed to reduce the particle size and break up hard aggregates that formed during the calcination process. The particle size reduction and breaking up of hard aggregates are advantageous for both the electrochemical performance and facilitating ion-exchange, as both require solid-state diffusion to proceed and by reducing the particle sizes, more surface area can be exposed to the electrolyte and exchange medium, as well as decreasing diffusion path lengths, aiding greater exchange.^31^ Compared to the pattern before the particle size reduction (Fig. S1), there were no observable differences in the phase purity or FWHM of the peaks, indicating that there was no crystallite size reduction and that the crystal structure was not damaged from the ball milling. As observed *via* SEM in Fig. 1d, even after milling, particles of ∼3–4 μm remained, but the average particle size was reduced to ∼1 μm, with an irregular and chunk-like morphology. EDS analysis (Fig. S2) showed a homogenous distribution of K, Ti and Nb with a ratio of 0.9 : 1 : 1.2, in line with previous results.^20^*Via* DLS (Fig. S3), the average particle size was determined to be 893 nm, corroborating the SEM. This is referred to as KTNO hereafter. To synthesize nickel ion-exchanged KTNO, the pristine KTNO was stirred in an aqueous NiCl_2_ salt solution of various ratios of Ni in solution to K in the KTNO. Using EDS, comparing the Ni to Ti ratio and the K to Ti ratio, the composition and thus the degree of exchange can be obtained. More precise inductively coupled plasma analysis could not be performed due to the high acid resistance of KTNO preventing dissolution.

By using the optimized conditions of 5 h, 80 °C and 0.25 M, the exchange could be finely tuned by varying the ratio of Ni^2+^ in the exchange solution to K^+^ in the KTNO, as shown in Fig. 1e. Two compositions were chosen to proceed with characterization, one high nickel content and one low nickel content, Ni_0.25_K_0.5_TiNbO_5_ and Ni_0.15_K_0.7_TiNbO_5_ and thus are designated Ni-KTNO-H and Ni-KTNO-L, respectively, with the contents chosen to investigate the exchange–performance relationship. The content of Ni can be further increased by subsequent rounds of exchange, but for the purposes of this work, the exchange was limited to one round. The XRD patterns of Ni-KTNO-H and Ni-KTNO-L are shown in Fig. 1f in comparison with the pristine KTNO. The growth of new phases was observed and was accompanied by a color change of the powder from white to green. No morphological changes were observed *via* SEM (Fig. S4). Full structure refinement could not be performed and thus atomic positions could not be extracted, as the ion exchange procedure introduces significant disorder and reduction in crystallinity, resulting in low quality patterns that result in overfitting and unstable refinements. However, changes in the lattice parameters could be estimated *via* a Le Bail fit, which are summarized in Table S1.^32^ For Ni-KTNO-H, this phase has a lower angle (002) peak than pristine KTNO (3.76° *vs.* 4.37°), corresponding to a *c* axis increase to 21.78 Å, indicating that the interlayer spacing has expanded to accommodate the Ni^2+^ and associated water molecules, as Ni^2+^ is smaller than K^+^ (0.83 Å *vs.* 1.33 Å) and thus should result in a *d*-spacing decrease, if solely intercalated. This phenomenon is termed the pillaring effect and has been covered extensively in the literature.^33–35^ The co-intercalation of water molecules is essential for increasing the amount Ni^2+^ that can be exchanged with K^+^, as by expanding the interlaying spacing, the exchange is able to penetrate into the bulk of the particle.^36^ Excluding the (011) and the (020) peaks, all other peaks were observed to decrease in relative intensity and broaden, indicating an introduction of strain, disorder and loss of crystallinity in those planes. The retention sharp (011) and (020) peaks indicates that the corrugated backbone, made up of TiO_6_ and NbO_6_ octahedra, is retained during the exchange. Furthermore, the retention of the (002) peak confirms that the layered structure is not lost. No changes to the XRD pattern were observed when stirring KTNO in deionized water, ruling out the intercalation of water molecules alone (Fig. S5). The XRD pattern of the Ni-KTNO-L sample revealed the presence of two distinct phases, as shown in Fig. 1f. Phase A retained the sharp, well-defined peaks of the pristine KTNO, with lattice parameters very similar to those of KTNO, while phase B exhibited broader peaks, matching well to the Ni-KTNO-H phase previously discussed. Phase A has its lowest angle peak at 4.31°, whereas phase B has its lowest angle peak at 3.69°, corresponding to a *c* axis of 18.702 Å and 21.36 Å, respectively, compared to 18.401 Å for pristine KTNO. Therefore, Ni-KTNO-L is bi-phasic with a proportion undergoing more exchange than the other. It is not a mixture of exchanged and unexchanged, as phase A still shows an increase in the *c*-axis when compared to pristine KTNO. Furthermore, it shows that the hydrated phases undergo distinct phase changes during exchange and are not solid-solution type reactions where the peaks shift to accommodate the exchanged ions.

**Fig. 2:**
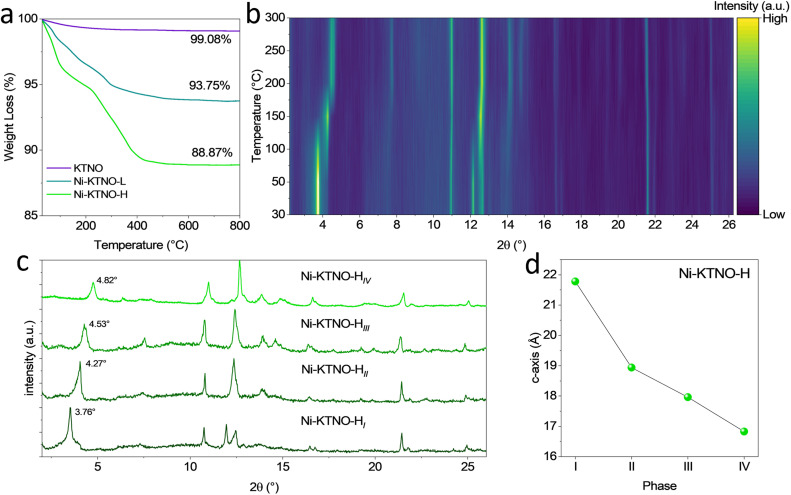
(a) TGA of KTNO, Ni-KTNO-L and Ni-KTNO-H up to 800 °C. (b) *in situ* variable temperature XRD between 30 °C and 300 °C. (c) individual patterns at 30 °C(i), 150 °C(ii), 300 °C(iii) and 400 °C(iv). (d) The change in the *c*-axis of phases I–IV.

The introduction of water molecules in the interlayer spacing is due to the exchange being performed in aqueous conditions, with the stability of this phase being an important factor in the electrode making process, which includes drying under vacuum. So, to quantify the degree of hydration present and the stability of the hydrated phase in Ni-KTNO-H and Ni-KTNO-L after drying at 60 °C, TGA analysis was performed. As shown in Fig. 2a, there was an 88.87 wt% decrease in weight for Ni-KTNO-H, compared to 93.75 wt% for Ni-KTNO-L. This allows the calculation of the number of associated water molecules, giving a composition of Ni_0.25_K_0.5_TiNbO_5_·1.77H_2_O and Ni_0.15_K_0.7_TiNbO_5_·0.96H_2_O, respectively. The dehydration mechanism is complex (Fig. S6), with several dehydration events occurring at 80 °C, 267 °C and 371 °C, with most of the weight loss occurring at the first stage. This presents an additional layer of complexity, as shown in previous studies, the co-intercalation of water molecules into layered structures expands the interlayer spacing significantly and can result in different electrochemical performance.^37^ To understand the dehydration process of the exchanged products, variable temperature *in situ* XRD was performed on Ni-KTNO-H up to 300 °C, which, combined with the TGA data, informs which phase can be best targeted for optimizing electrochemical performance. As can be seen from Fig. 2b, between 30 and 300 °C for Ni-KTNO-H there is a two-step dehydration process, one between 50 and 100 °C and one between 150 and 200 °C, with the individual patterns at these temperatures shown in Fig. 2c, along with the final dehydration which was performed *ex situ* at 400 °C. This differs marginally from the TGA data, but it is commonly observed that the heating rate can significantly affect the exact temperature of dehydration and as the TGA ramp rate was faster than the *in situ* XRD, the differences can be considered reasonable.^38,39^ A Le Bail fit was applied to each pattern to estimate the lattice parameter changes (Table S1), as a full refinement was not possible due to the low crystallinity of the materials. For phases II, III and IV, the (002) peak decreases to 4.27°, 4.53° and 4.82°, corresponding to the *c* axis decreasing to 18.93, 17.96 and 16.8 Å, respectively, as shown in Fig. 2d, as the water molecules holding the layers apart are removed. The *c*-axis shrinks below the pristine KTNO as the larger K^+^ has been replaced with the smaller Ni^2+^. The layers contract as the water molecules that are acting as pillaring species are removed. The four phases with different levels of hydration would be expected to perform differently due to the differences in interlayer spacing and water content and this must be kept under careful consideration and will be investigated in a later section.

**Fig. 3:**
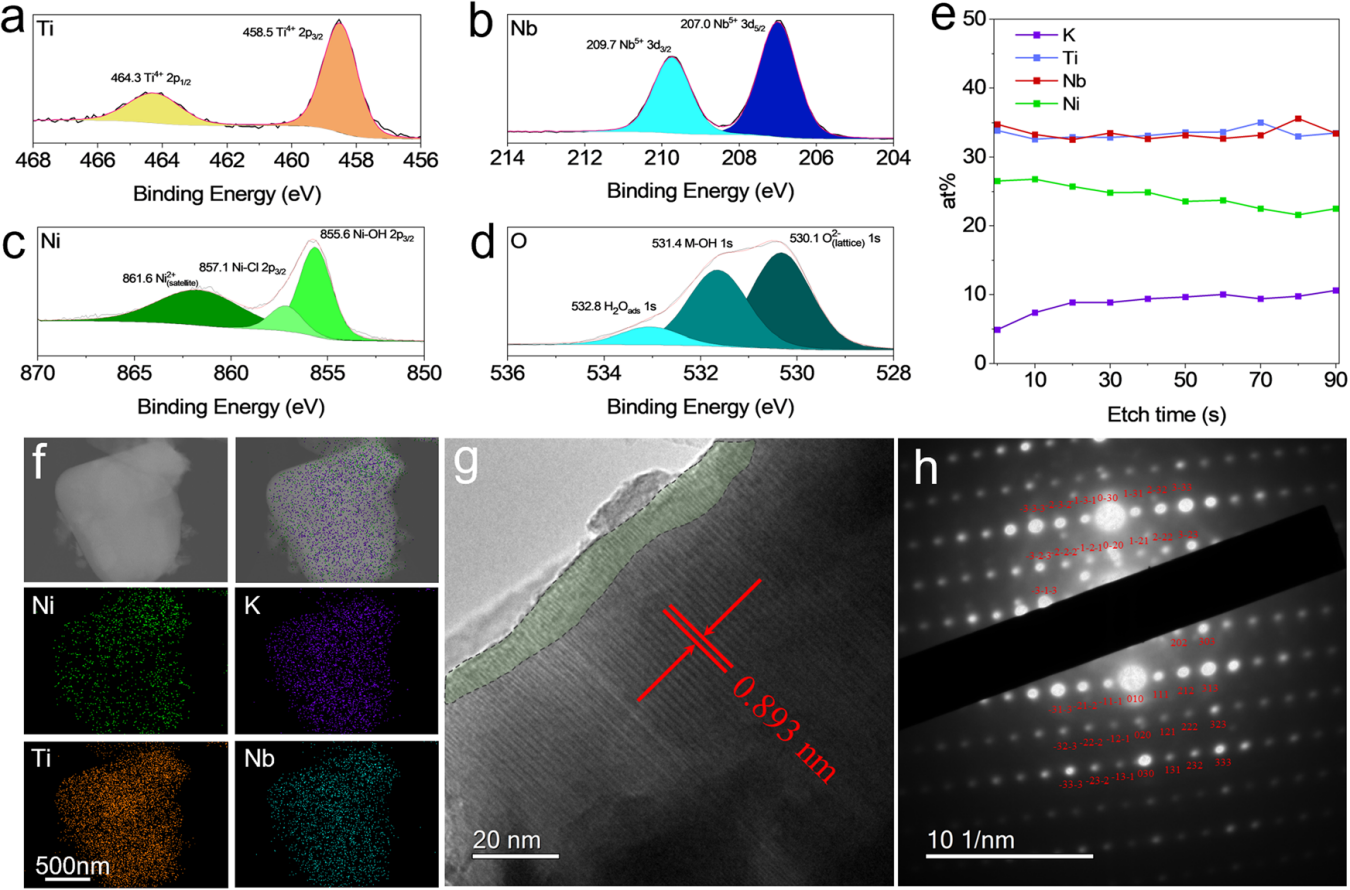
High resolution XPS spectra of (a) Ti, (b) Nb, (c) Ni and (d) O for Ni-KTNO-H. (e) abundance of elements during depth profile for Ni-KTNO-H from 0 s to 90 s etching. (f) STEM-EDS image of Ni-KTNO-H. (g) HRTEM image of Ni-KTNO-H showing the (002) lattice plane. (h) SAED pattern of the Ni-KTNO-H bulk, obtained down the [101] zone axis.

To examine the surface composition of the materials, XPS analysis was performed. Due to the ultra-high vacuum that XPS is performed under, the water molecules are expected to be pulled from the interlayer spacing, dehydrating the sample and preventing analysis of the hydrated phase under these conditions. However, the results are still transferable to both the partially hydrated and fully hydrated phases, as the surface composition is unlikely to change significantly. XPS for the pristine KTNO (Fig. S7) shows that the Ti and Nb are in their highest oxidation state of 4+ and 5+, respectively, with the Ti 2p_3/2_ and Nb 3d_5/2_ peaks located at 458.3 and 206.9 eV, respectively. The elemental composition from the survey was 1 : 0.86 : 1 (K : Ti : Nb), corroborating the EDS results (Fig. S8). For Ni-KTNO-H, as shown in Fig. 3a and b, the Ti 2p_3/2_ and Nb 3d_5/2_ peaks appear at 458.5 and 207.0 eV, respectively, showing that the ion-exchange process did not affect the initial oxidation state of either Ti or Nb. *Via* the survey spectrum (Fig. S9), the ratio of Ni/Ti was determined to be 0.78 : 1, indicating that there are some Ni species adsorbed onto the surface of the Ni-KTNO particles, forming a Ni-rich layer. Despite this, both the Ti and Nb were detected even with no etching, showing that the Ni rich layer was below 10 nm thick as XPS detection depth is up to 10 nm, which rules out the possibility of the Ni only being concentrated on the surface with no bulk exchange.^40^ The Ni 2p peak, as shown in Fig. 3c, was detected to have a large component of Ni–OH groups at 855.6 eV, which are an inherent feature of metal oxides exposed to aqueous conditions and have been extensively documented.^41,42^ This was confirmed by the high resolution O 1 s spectra, which required a significant M–OH component at 531.4 eV to fit adequately, as shown in Fig. 3d.^43^ A component of Ni–Cl was required to fit the spectra sufficiently with a peak at 857.1 eV. This was confirmed by a small amount of chlorine detected, despite thorough washing, but was not stoichiometric with the amount of detected nickel (Fig. S9). This can be explained by some of the Ni^2+^ ions on the surface retaining a Cl^−^ ion after replacing a K^+^, as it was still exposed to a high concentration of Cl^−^ ions in solution. In addition, a small amount of potassium was detected (4.8 at%) without etching. This could originate either from structural K^+^ that is not exchangeable, or from some reverse exchange, as the K^+^ ions deintercalated are still present in the solution. To examine how the composition varies with depth for Ni-KTNO-H, etching was performed, with the results shown in Fig. 3e. As the etching depth increased, the contribution of K increased, from 4.9 at% at the surface to 10.6 at% after 90 s of etching. The Ni contribution reduced alongside, decreasing from 26.5 at% to 22.5 at% after 90 s. Excluding factors such as preferential removal of lighter atoms, there is a clear gradient in composition in the particles, showing that exchange is more prevalent at the surface of the particles, but is still occurring deeper into the bulk. To examine the crystallinity and chemical composition at the near surface, TEM and STEM-EDS were performed on Ni-KTNO-H. Performing STEM-EDS as shown in Fig. 3f, reveals that the Ni is present throughout the particle, but has a slight concentration on the surface, corroborating the XPS, TEM and EDS results previously gathered. As shown in Fig. 3g, the particle is still highly crystalline, with a *d* spacing of 0.893 nm, corroborating the XRD pattern, which also shows a *d*-spacing of 0.897 nm for the (002) of Ni-KTNO-H_III_. This indicates that the water was also removed in the high vacuum of the TEM. There is a difference between the surface of the particle and a few nm into the particle, which clarifies the XPS results and indicates there is a layer on the surface of the Ni-KTNO particles that is particularly Ni rich. This may be a benefit for the capacitive contribution to charge storage, as Ni-based materials are well known to show pseudocapacitive behavior.^44^ However, this layer is relatively thin and further shows that the surface modification is not extensive, ruling out the possibility that the Ni is wholly concentrated on the surface with little penetration into the bulk. This surface *vs.* bulk exchange is well understood in the ion-exchange literature, as initial exchange at the surface with intercalation of associated water molecules can induce interlayer spacing expansion that allows for diffusion of the exchange ion deeper into the particle.^45^ The high crystallinity of the particles was further confirmed *via* the SAED pattern, as shown in Fig. 3h. Quantification of this diffraction pattern indicates that the zone axis shown is the [101] orientation, with lattice parameters well matching the lattice parameters obtained from the previous Le Bail fit of the Ni-KTNO-H_III_ phase (Table S1).

**Fig. 4:**
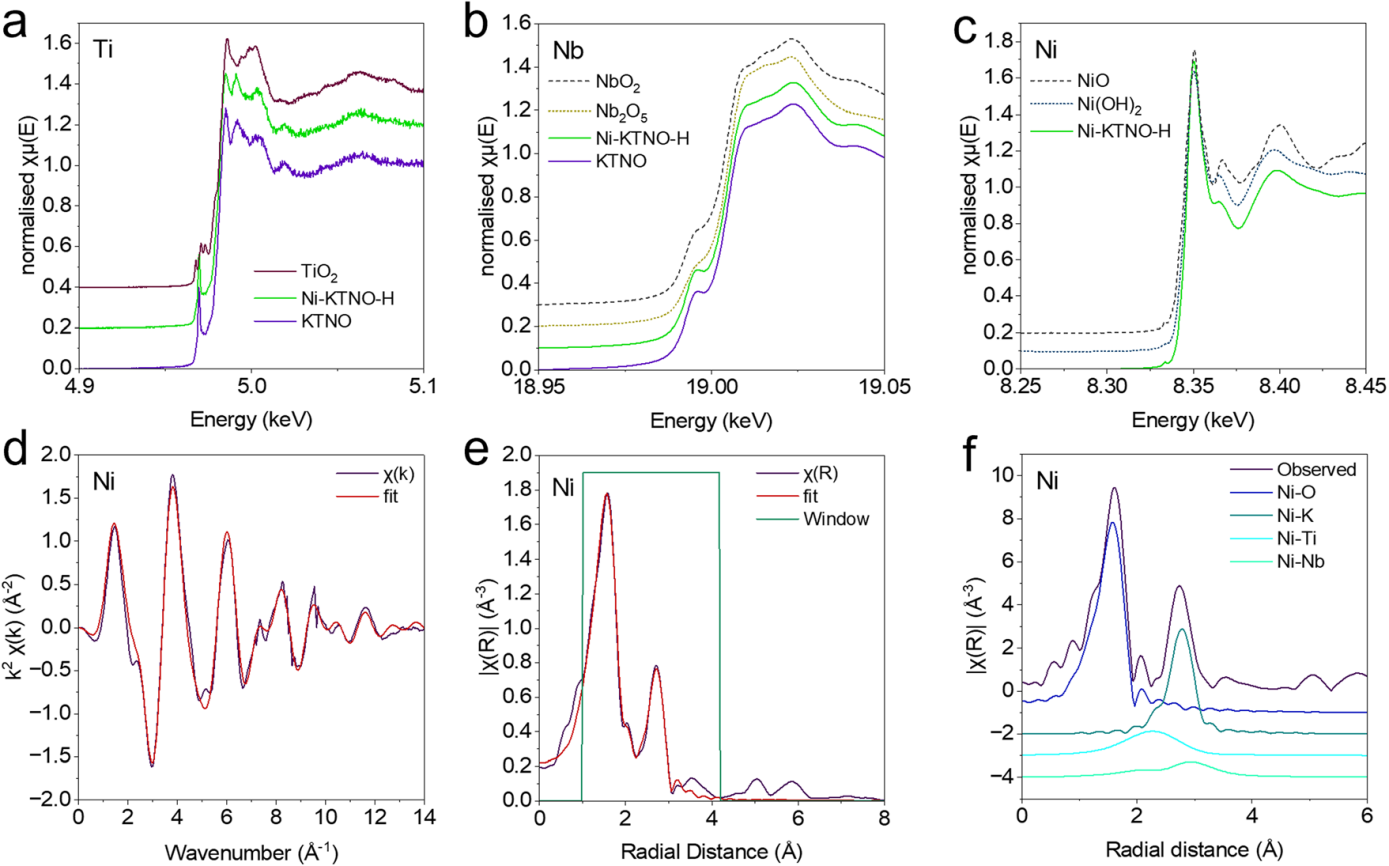
(a) Ti K-edge XANES spectra. (b) Nb K-edge XANES spectra. (c) Ni K-edge XANES spectra. (d) Ni K-edge in *k* space. (e) Ni K-edge in R space. (f) Components of fit of *χ*(R).

The local structure of Ni-KTNO-H and the pristine KTNO was then investigated *via* XAS. As shown in Fig. 4a, the Ti K-edge position shows no changes between the samples, indicating that no change in the valence of the Ti occurred during the exchange, with the edge position at 4981.7 and 4981.8 eV for KTNO and Ni-KTNO, respectively. However, one notable change is that the pre-edge peak located at 4968.9 eV is slightly depressed for Ni-KTNO *vs.* KTNO, indicating that the Ti octahedral coordination has been slightly distorted during the exchange, as the pre-edge peak is highly sensitive to the local geometry, distortions and Ti–O bond lengths.^46,47^ Similarly, for the Nb K-edge shown in Fig. 4b, there was no change in edge position after exchange, with the measured KTNO and Ni-KTNO edge energy at 18994.4 and 18994.5 eV, respectively. This demonstrates that to incorporate the Ni^2+^ ion, K^+^ must be exchanged and removed to maintain charge neutrality, as neither the Nb nor Ti changed valence in compensation. EXAFS analysis was performed on the Ni K-edge data as shown in Fig. 4c, with a basic first-shell fitting, with the *χ*(k) and *χ*(R) shown in Fig. 4d and e, respectively. This provides a reasonable fit with a reduced *χ*^2^ of 98.46 and an R-factor of 0.00784. The extracted coordination number, bond length and *σ*^2^ are shown in Table S2. The lengths of the extracted Ni–Ti and Ni–Nb bond lengths, Fig. 4f, 2.84 and 3.13 Å respectively, suggest that the Ni^2+^ preferentially coordinates in the octahedral site in the interlayer spacing closest to the TiO_6_ unit, rather than a random distribution over the sites. This is implied by the degree of exchange, as the maximum achieved under the conditions was ∼50%, that the unexchangeable K^+^ is more tightly bound to the octahedral site situated close to the NbO_6_ unit.

### Electrochemical performance

**Fig. 5:**
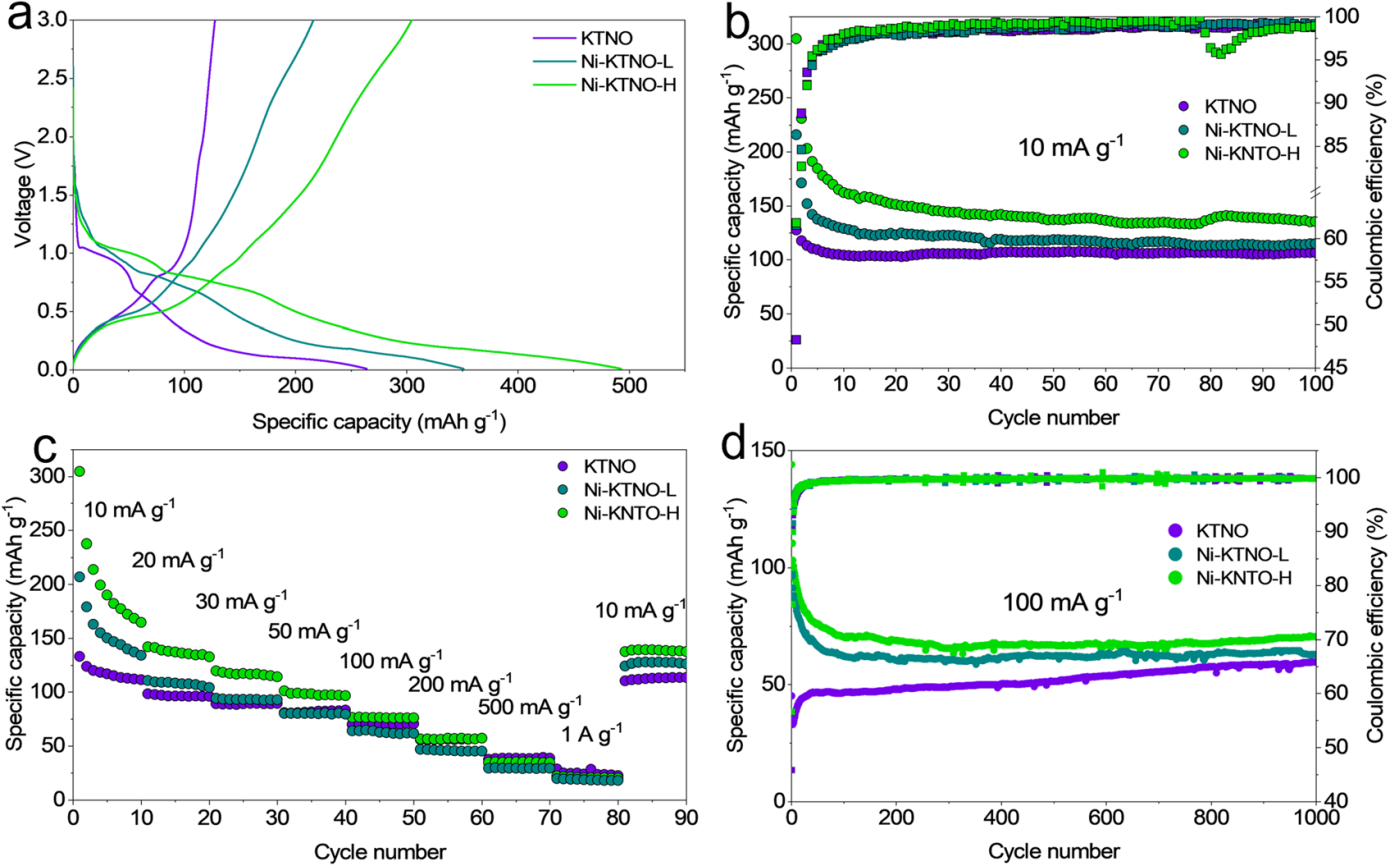
(a) The first galvanostatic charge–discharge curves of KTNO, Ni-KTNO-L and Ni-KTNO-H at 10 mA g^−1^. (b) Long cycling performance of KTNO, Ni-KTNO-L and Ni-KTNO-H at 10 mA g^−1^ (c) Rate performance of KTNO, Ni-KTNO-L and Ni-KTNO-H from 10 mA g^−1^ to 1 A g^−1^, using the charge capacity. (d) Long cycling performance of KTNO, Ni-KTNO-L and Ni-KTNO-H at 100 mA g^−1^.

To investigate the electrochemical performance and exchange–performance relationship of the ion-exchanged samples, Ni-KTNO-H and Ni-KTNO-L were electrochemically tested in comparison to pristine KTNO. As mentioned previously, the level of hydration is a key factor for electrochemical performance. As the electrode drying procedure requires 60 °C under vacuum overnight, the Ni-KTNO-H_I_ phase would dehydrate under these conditions and thus cannot be tested, so the partially hydrated phase Ni-KTNO-H_II_ is initially measured and will be referred to as Ni-KTNO-H. The galvanostatic charge/discharge profiles under constant current control of 10 mA g^−1^ are displayed in Fig. 5a. The potassium storage performance of the pristine KTNO was in line with previous results, achieving a first charge capacity of 127.5 mAh g^−1^ with an initial coulombic efficiency (ICE) of 48.3%.^20^ In contrast, the Ni-KTNO-L and Ni-KTNO-H displayed a specific charge capacity of 216.1 and 304.6 mAh g^−1^, respectively, during the first cycle, which corresponds to a 170 and 240% increase in capacity compared to KTNO. The high voltage capacity seen in the first discharge consists of the initial pseudocapacitive intercalation, along with significant SEI formation, as it typical for TMO negative electrode materials.^48,49^ The profiles also show some notable differences, with both the Ni-KTNO samples having significantly more capacity above 1 V, 151 and 103 mAh g^−1^ for Ni-KTNO-H and Ni-KTNO-L, respectively, compared to only 27 mAh g^−1^ for KTNO. The cause of this increased capacity above 1 V is threefold. Firstly, the difference in site energy from the large expansion of the interlayer spacing caused by the co-intercalated water molecules and Ni^2+^, results in differences in electrostatic interactions between the “structural” ions and the intercalated ions, leading to a greater amount of K^+^ intercalated over a much wider voltage range. Secondly, the Ni rich surface layer has an enhanced pseudocapacitive contribution, providing more charge storage, as Ni-based pseudocapacitors are common in the literature.^44,50^ Thirdly, while Ni^2+^ has a higher charge and electronegativity than K^+^, charge balancing requires one Ni^2+^ to replace two K^+^, increasing the amount of void space within the crystal structure and allowing for more K^+^ to be intercalated, while introducing potassium vacancies that can be filled during intercalation. While the change in surface composition undoubtedly assists pseudocapacitive storage, the increase in capacity is unlikely to be solely additional surface charge storage, as the samples did not undergo significant morphological change during ion-exchange. In terms of structural stability, the co-intercalated water molecules appear to negatively affect cycle life indirectly, as by increasing the intercalation of K^+^, this may increase the strain on the lattice and thus result in greater structural breakdown, hence the rapid capacity fade in the first cycles. This may not be detectable by XRD, which relies on the crystallinity of the sample to operate. The phenomenon of water-mediated stability is well documented in other fields and is a promising area of future work for this material.^51,52^

Ni-KTNO-H also shows an enhanced semi-plateau around 0.5 V, demonstrating that the greater the degree of exchange, the more the intercalation reaction is enhanced. One important aspect of Ni-KTNO is its stability to reverse the ion-exchange. If Ni-KTNO is placed in a potassium rich salt solution, like the electrolyte of a cell, it could be possible to reverse the ion-exchange and return to KTNO. This was tested by immersing an electrode in electrolyte in an AMPIX cell, then measuring the XRD pattern over 24 h. The XRD pattern was unchanged over that period (Fig. S10), indicating that the ion-exchange was not reversible at room temperature and under the conditions present in the cell at rest. Furthermore, it was confirmed that the ion-exchange is stable under electrochemical conditions. EDS analysis was performed during the initial charge and discharge and then after 1000 cycles. While the precision of EDS is lower than other techniques like ICP-MS and XPS, it is sufficient to examine the elemental composition in the context of the charge–discharge process. The ratio of Ni/Ti remained constant over the first charge and discharge and even after 1000 cycles (Table S3), showing the exchange is stable and Ni is not deintercalated, even after extensive cycling. This is a vital part of the applicability of the ion-exchange technique, because if the deintercalation of the exchange ion during electrochemical cycling could cause unwanted side reactions or even poison the electrolyte and counter electrode, thereby degrading performance. The cycling performance of each material was measured at 10 mA g^−1^ to determine its low rate stability. As shown in Fig. 5b, at 10 mA g^−1^, there is a significant drop in capacity over the first few cycles for both of the Ni-KTNO samples, with the Ni-KTNO-H and Ni-KTNO-L retaining 162.2 and 128.8 mAh g^−1^ by cycle 10, 53.2% and 59.6% of the initial charge capacity, respectively. If this large capacity fading can be addressed and stabilized, this would represent a significant performance milestone for TMO negative electrode materials. After this initial rapid drop, the rate of decrease slowed dramatically and after 100 cycles, Ni-KTNO-H and Ni-KTNO-L retained 135.4 and 115.0 mAh g^−1^, 44.4% and 53.2% of the initial capacity, respectively. When compared to the pristine KTNO, which retained 104.2 mAh g^−1^ and 106.6 mAh g^−1^ by cycle 10 and 100, respectively, there is a clear tradeoff between capacity and cycling stability. This is unsurprising, as the greater intercalation puts a greater degree of stress on the crystal structure and is therefore more likely to suffer from degradation processes that negatively impact cycling stability, particularly at the more extreme conditions that low-rate cycling provides. However, after the initial capacity fade, no further instability of the material is observed, indicating that the pillaring water molecules are not adversely affecting the structural stability of Ni-KTNO-H during long cycle procedures. The rate performance of KTNO, Ni-KTNO-L and Ni-KTNO-H is shown in Fig. 5c, with Ni-KTNO-H and Ni-KTNO-L outperforming KTNO at all rates until 200 mA g^−1^, after which all the samples performed equally. This indicates that the Ni^0^/Ni^2+^ redox couple is kinetically limited and is more active at lower rates. As shown in Fig. 5d, under the 100 mA g^−1^ cycling, the benefit of the ion-exchange becomes clearer, as the pristine KTNO requires 40 cycles to activate and with the capacity still increasing slowly over 1000 cycles, retaining 59 mAh g^−1^. This is not present in the exchanged samples, showing that the exchange improves the kinetics involved with the intercalation–deintercalation process. For the Ni-KTNO samples, there is still a rapid drop in capacity, similar to the 10 mA g^−1^ rate, with both stabilizing after around 100 cycles, with Ni-KTNO-H and Ni-KTNO-L retaining 70.4 and 62.9 mAh g^−1^ after 1000 cycles, 48.9 and 54.1%, respectively.

To investigate the role that hydration plays in the electrochemical performance of Ni-KTNO, the fully dehydrated phase Ni-KTNO-H_IV_ was also electrochemically tested (Fig. S11). Ni-KTNO-H_IV_ shows lower initial performance (160 mAh g^−1^), but is much more stable, retaining the initial performance after returning to 10 mA g^−1^ and maintaining this capacity when further cycling. This clearly shows that the water molecules expanding the interlayer spacing are vital for the enhanced electrochemical performance that is observed in Fig. 5a, but even with their exclusion, the performance of the dehydrated Ni-KTNO exceeds the pristine KTNO. To further illustrate the applicability of this ion-exchange procedure to other types of ion-batteries, Ni-KTNO-H and KTNO were tested in NIBs. The capacity of the pristine KTNO showed good performance in NIBs (Fig. S12), with a peak discharge specific capacity of 95.2 mAh g^−1^ at 10 mA g^−1^ (excluding the first cycle), indicating that KTNO is also able to store Na^+^. The charge–discharge curves are also conducive for achieving high energy density with less risk of sodium plating (Fig. S13). In contrast, the Ni-KTNO-H performance was significantly enhanced *vs.* KTNO, achieving 273.1 mAh g^−1^ in the first charge cycle at 10 mA g^−1^, a 322% increase. The charge–discharge curves of Ni-KTNO-H show similar features to KIBs, with the capacity increase spread over a wide voltage range (Fig. S14). Furthermore, the rate performance in NIBs showed a very similar pattern to KIBs, where Ni-KTNO-H decreased in performance with increasing rate until at parity with KTNO (Fig. S12), indicating that enhanced intercalation is responsible for the increase in capacity, as intercalation decreases more significantly with higher rates than capacitive storage. The performance in NIBs demonstrates that the process of ion-exchanging can be transferred to different battery chemistries, potentially opening the door to further enhancing mature materials like LTO and NTO.

### Kinetics of electrochemical reaction

**Fig. 6:**
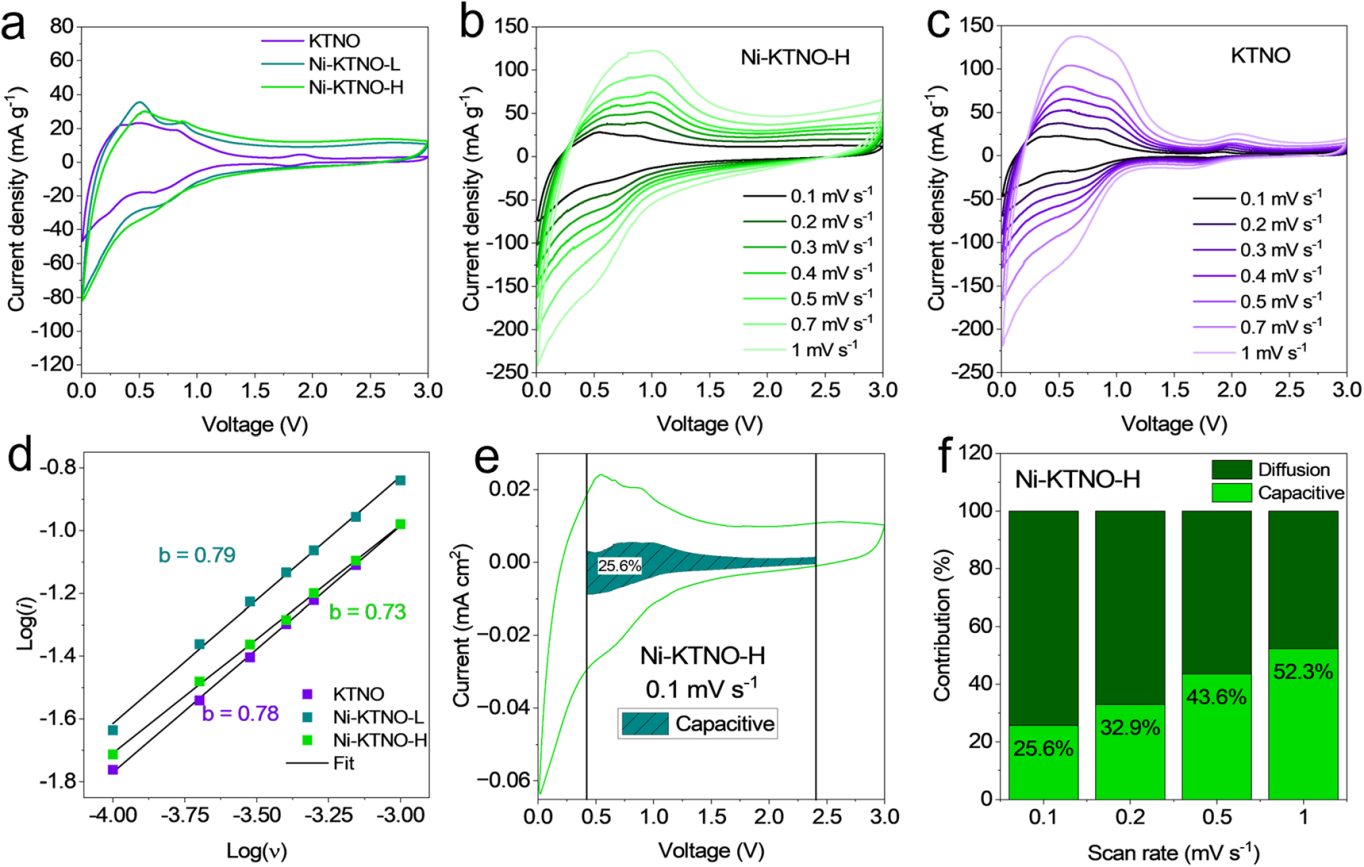
(a) CV curves of KTNO, Ni-KTNO-L and Ni-KTNO-H. (b) CV curves of Ni-KTNO-H at scan rates from 0.1 to 1 mV s^−1^. (c) CV curves of KTNO at scan rates from 0.1 to 1 mV s^−1^. (d) calculated b values for KTNO, Ni-KTNO-L and Ni-KTNO-H. (e) Capacitive contribution for Ni-KTNO-H at 0.1 mV s^−1^. (f) The ratio between the capacitive and diffusion capacitive contribution for Ni-KTNO-H at different scan rates.

To examine the reversibility and the kinetics of the electrochemical reaction, cyclic voltammetry (CV) was performed on KTNO, Ni-KTNO-L and Ni-KTNO-H, from 0.1 mV s^−1^ to 1 mV s^−1^. As shown in Fig. 6a, the KTNO CV features one set of redox peaks centered at 1.86 V and one set centered at 0.79 V. In contrast, Ni-KTNO-H and Ni-KTNO-L no longer feature the redox peak at 0.79 V and have an enhanced current response at all voltages, suggesting that the ion-exchange has widened the intercalation potential and allows for greater intercalation. The current density recorded at 0.79 V for Ni-KTNO-H is also larger than either of the other two samples, reaching 23.8 mA g^−1^ compared to 23.2 and 22.9 mA g^−1^ for Ni-KTNO-L and KTNO, respectively. CVs were gathered at 0.1, 0.2, 0.4, 0.5, 0.7 and 1 mV s^−1^ for Ni-KTNO-H, Ni-KTNO-L and KTNO, as shown in Fig. 6a, b and S15, respectively. From these, using the power law eqn S2, the *b-*values for the three samples could be calculated, using the peaks centered at 0.79 V. As shown in Fig. 6d, the *b*-values ranged from 0.73 for Ni-KTNO-H, 0.79 for Ni-KTNO-L and 0.78 for KTNO, which fall between the ideal values of 1 for a capacitor and 0.5 for an intercalation material, so these materials show features of pseudocapacitive intercalation.^44^ When analyzing the shape of the CV profiles, it has numerous features that are indicative of pseudocapacitive intercalation. Firstly, while there are obvious redox peaks, they are broad and not particularly intense.^53^ This rules out purely capacitive behavior, as it lacks the classic box shape of true capacitors, but also does not follow full faradaic battery-like behavior that produces a sharp redox peak with a corresponding phase change. Secondly, the large particle size of the samples excludes any capacitive effects that may arise from nanostructuring that is present in many nanomaterials, as the particles are on average 1 μm.^54,55^ Thirdly, the *b* values calculated indicate a pseudocapacitive controlled process, around 0.75. To quantify the amount of charge storage controlled by capacitive and diffusion-controlled processes, the Dunn method was used, between the limits of 0.415–2.4 V.^56^ As shown in Fig. 6e for Ni-KTNO-H, at 0.1 mV s^−1^, much of the current response follows intercalation-based behavior, with only 25.6% calculated to arise from capacitive processes. As the scan speed increases, Fig. 6f, the capacitive contribution increases correspondingly, with 32.9%, 43.6% and 52.3% at scan speeds of 0.2, 0.5 and 1 mV s^−1^, respectively. In contrast, the capacitive contribution for Ni-KTNO-L and KTNO was calculated to be higher at 31.2 and 55.5% at 0.1 mV s^−1^, respectively (Fig. S16 and S17), while also having higher capacitive contributions at higher scan rates (Fig. S18 and S19). This demonstrates that the exchange of Ni for K within the KTNO structure greatly enhances the intercalation of K^+^, a diffusion-controlled process. Furthermore, on the anodic scan, the area above 1 V corresponding to the long sloping area in the GCD curves exhibits high diffusion control, indicating that deintercalation of K^+^ occurs over a much wider range than in the pristine KTNO.

**Fig. 7:**
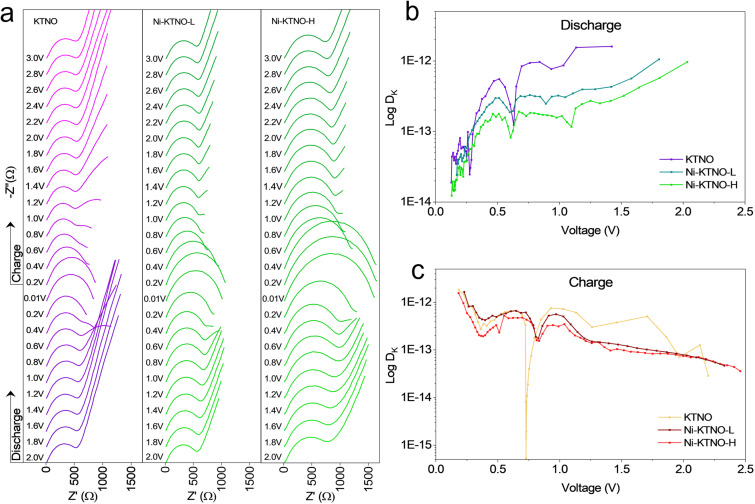
(a) *In situ* EIS of KTNO, Ni-KTNO-L and Ni-KTNO-H, discharge from 2 V to 0.01 V, then charging to 3 V. (b) Calculated *D*_K_ values during discharge for KTNO, Ni-KTNO-L and Ni-KTNO-H. (c) Calculated *D*_K_ values during charge for KTNO, Ni-KTNO-L and Ni-KTNO-H.

Examining the interfacial charge transfer kinetics of the samples, *in situ* EIS was performed after 3 cycles of charge and discharge to stabilize the materials. As shown in Fig. 7a, the initial Nyquist plots show that the overall impedance for the Ni-KTNO-H is higher than either the KTNO or Ni-KTNO-L, with an *R*_ct_ of 743 Ω *vs.* 454 and 488 Ω, respectively, as fitted using the equivalent circuit shown in Fig. S20. This can be attributed to the Ni-rich layer on the Ni-KTNO-H, as much more of the potassium was exchanged in the near-surface sites, which results in an increase in the impedance for potassium charge transfer. The measured diffusion impedance deviates from the semi-infinite Warburg impedance that occurs at 45° on the Nyquist plot, which is another strong indicator of pseudocapacitance.^44,57^ During the charge–discharge cycle, very similar behavior is observed for all samples, where the impedance rises as the material is potassiated and then decreases when de-potassiated. At the end of discharge the R_ct_ values for Ni-KTNO-H, Ni-KTNO-L and KTNO are 1584, 1012 and 806 Ω, respectively, when the materials become fully potassiated with all the active sites filled, thus the impedance associated with the interfacial charge transfer starts to rise and the diffusion into the structure decreases, as shown by the reduction of diffusion impedance coefficient. As Ni-KTNO-H has the largest increase in intercalation, it also exhibits the largest increase in *R*_ct_. When fully charged, the *R*_ct_ of all the samples decreases back to close to the initial starting values, showing the reversibility of the charge–discharge process. To examine the differences in diffusion of K^+^ ions within the ion-exchanged material *vs.* pristine, galvanostatic intermittent titration technique (GITT) was performed for KTNO, Ni-KTNO-L and Ni-KTNO-H (Fig. S21–S23), allowing for an estimate of the diffusion coefficient (*D*_k_) *via* eqn S3.^58^ As shown in Fig. 7b, the average *D*_k_ values during discharge for KTNO were observed to be slightly higher than Ni-KTNO-L and Ni-KTNO-H. This suggests that the exchange of K^+^ for Ni^2+^ in the interlayer spacing negatively affects ionic mobility, and the positive effect of introducing K^+^ vacancies is not fully balanced out by the lower mobility of Ni^2+^ in the channels, resulting in overall lower ionic mobility. *D*_k_ decreases as the samples are potassiated, which indicates the intercalated K^+^ ions occupy previously available sites for diffusion, disrupting ionic mobility. In addition, the additional K^+^ ions result in greater electrostatic repulsion, reducing mobility and is commonly observed in the literature.^49,59,60^ This corroborates the EIS results, which also suggests that intercalating more K^+^ becomes less favorable at higher states of charge. There are sharp drops in ionic diffusion around 0.6 V, which has been understood in the literature to signify a phase transformation.^61^ During charge, all three samples performed similarly, but the KTNO experienced a much sharper drop in *D*_k_ value than Ni-KTNO-H or Ni-KTNO-L at 0.73 V.

### Charge storage mechanism

**Fig. 8:**
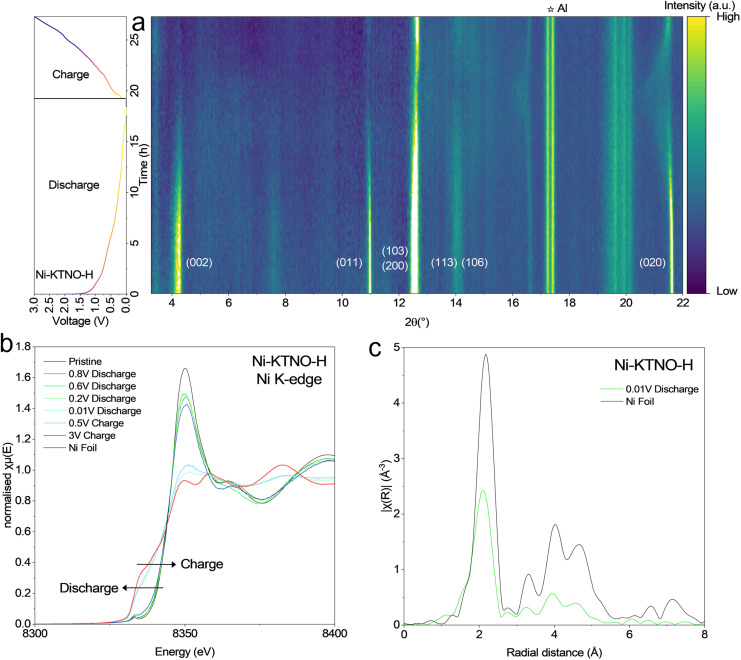
(a) In-*operando* XRD patterns of freestanding Ni-KTNO-H electrode, at 15 mA g^−1^. (b) Ni K-edge *ex situ* XANES spectra during discharge and charge. (c) Ni K-edge *ex situ* XANES spectra of the fully discharged sample plotted in R space and compared to Ni foil.

To understand the mechanism of charge storage, a combination of techniques was employed, including in-*operando* and *ex situ* XRD to allow identification of phase changes of Ni-KTNO as it undergoes potassiation/depotassiation and *ex situ* XAS to observe any changes to the oxidation state and local environments of the Ni. The in-*operando* XRD patterns of Ni-KTNO-H when discharging to 0.01 V, then charging to 3 V are shown in Fig. 8a. As observed, there is no detectable change to the Ni-KTNO-H XRD pattern above 0.4 V during discharging, as there are no peak shifts or new phases present. This implies that the mechanism of charge storage in that voltage range is pseudocapacitive, with fast redox reactions occurring near or at the interface between the particles and the electrolyte, along with SEI formation as the first cycle is measured. However, when discharging below 0.4 V, there is a clear phase change, with a clear reduction in intensity of the (002), (011) and (020) planes and the (200) and (013) merging to one lower angle peak, at the same 2*θ* position as Ni-KTNO-H_III_. This is corroborated by both the *in situ* EIS and GITT measurements, which both show changes to the *R*_ct_ and *D*_k_ values, respectively, between 0.6 and 0.4 V. The reduction of the (002) plane indicates that stacking disorder has been introduced into the layers because of the increased K^+^ intercalation, whereas the shift of the (200) and the (013) indicates that the phase is likely to have dehydrated during the discharge. The loss of the (002) was confirmed *via ex situ* XRD (Fig. S24). Charging back up to 3 V does not result in a reversible phase transformation, with the fully discharged phase remaining, further implying that phase change is dehydration. One avenue for improvement is stabilizing water molecules in the interlayer spacing, possibly through subsequent ion-exchanges with other ions, which might retain the high capacity of the initial cycles. In the *ex situ* XRD, however, some small proportion of the (002) peak was observed to reform at 4.05°, indicating that the loss of the (002) is reversible and is likely below the signal to noise limit of the laboratory XRD, which is further reduced by the additional components of the cell. This lower (002) peak implies that the full extraction of K^+^ was not possible, with the unextractable K^+^ expanding the interlayer spacing. To investigate the long-term structural stability of Ni-KTNO-H, *ex situ* XRD was performed after 1000 cycles at 100 mA g^−1^. As shown in Fig. S25, the Ni-KTNO-H structure remains intact, with the (002) peak remaining at 4.33° and retains the pristine phase II structure. This differs from the in-*operando* measurement because the amount of intercalated K^+^ at higher rate is reduced, likely reducing the phase transformation and loss of water. Note that there could be a small level of amorphization of the materials over the course of 1000 cycles, which would be undetectable *via* XRD.

*Ex situ* XAS on the Ni K-edge was performed at 0.8, 0.6, 0.2 and 0.01 V during the discharge process and at 0.5 and 3 V during the charge process. The Ni K-edge showed a significant change in both the edge position and the EXAFS spectra after discharging below 0.2 V, Fig. 8b, with the edge shifting from 8346.2 to 8344.6 eV, indicating that it had been reduced to compensate for the intercalated K^+^. In addition, the FT EXAFS spectra *χ*(R) shows a major component that matches well to Ni–Ni metal bonds, Fig. 8c. This would imply that during K^+^ intercalation, the Ni^2+^ is reduced to Ni^0^, forming Ni metal nanoclusters in the interlayer spacing. Our results differ from previous results, that observed an alloying reaction, thus the mechanism by which the electrochemical performance was enhanced was an increase in intercalation. This is further evidenced by the differing electrochemical performance of Ni-KTNO in NIBs, which is far higher than reported values.^28^ Furthermore, the observation of a NiK alloy outside of high pressure environments is extremely rare with many examples of Ni foam being used in negative electrode applications with no observation of NiK alloy, so is unlikely to contribute in this case.^62,63^ This process is partially reversible, as charging up to 3 V results in the spectra matching well to the pristine, as shown in Fig. 8b, with the oxidation occurring between 0.5 and 3 V but the edge jump does not shift all the way back, remaining at 8346.0 eV (Fig. S26). This is further confirmed as after 5 cycles, the edge shifts to 8344.3 eV at full discharge and back to 8346.3 eV at full charge, so some slight portion of the redox activity of the Ni is lost after the first cycle. There was no observable change in the XANES or EXAFS of the Nb or Ti K-edge during discharge or charge (Fig. S27 and S28). This is surprising given the electrochemical performance of 492.8 mAh g^−1^ in the first discharge (includes SEI formation) then 309.2 mAh g^−1^ in the first charge, 2.9 K^+^ should be intercalated and de-intercalated per formula and there must be charge compensated by the reduction of the Ti and/or Nb. Examining this will be the subject of future work.

## Conclusion

To conclude, *via* a facile ion-exchange procedure, Ni_0.25_K_0.5_TiNbO_5_ was successfully synthesized and characterized as a negative electrode material for KIBs. The K^+^ initially present in the structure was shown to be exchangeable with Ni^2+^, along with co-intercalated water molecules, resulting in a highly expanded interlayer spacing. The water molecules act as pillaring species, holding the transition metal oxide layers apart, but can be easily removed, resulting in distinct hydrated phases and a subsequent contraction of the *c*-axis. By targeting specific phases and degrees of Ni^2+^ exchange, the electrochemical performance can be tuned. The optimum phase, Ni-KTNO-H_II_, exhibited a specific capacity of 304 mAh g^−1^ on the first charge, a 240% increase over the pristine KTNO, but dropped to 135 mAh g^−1^ at cycle 100. A fruitful research direction for this material would be stabilizing this initial performance, potentially through subsequent rounds of ion-exchange. The mechanism of charge storage was investigated, with a clear phase transition observed below 0.4 V *vs.* K/K^+^, but was coupled with a dehydration of the Ni-KTNO phase. The most important finding was that, through *ex situ* XAS analysis, the Ni present in the material was shown to undergo a reversible Ni^2+^/Ni^0^ redox couple during discharge and charge, something that to the author's knowledge, has not been demonstrated before in the KIB literature. These results demonstrate the potential for the ion-exchange of redox active species, as with continued development, this technique could be applied to many commercially relevant negative electrode materials.

## Author contributions

C. A. F. N.: conceptualization, methodology, formal analysis, investigation, visualization, writing – original draft, writing – review & editing; A. P. V. K. S.: methodology, formal analysis, investigation; W. R., Y. M., A. S., Y. H., Y. L, and J. A. G.: investigation, formal analysis; T. I. H: methodology, investigation, resources; V. C.: investigation, resources; G. S.: methodology, investigation, formal analysis, writing – review & editing; Y. X.: conceptualization, investigation, project administration, supervision, funding acquisition, writing – review & editing.

## Conflicts of interest

There are no conflicts to declare.

## Supplementary Material

SC-OLF-D5SC04984A-s001

## Data Availability

Data for this article is available from the corresponding authors upon reasonable request. The data supporting this article have been included as part of the SI. See DOI: https://doi.org/10.1039/d5sc04984a.
