# Supplementary material for: Enhanced ion intercalation in NixK1−2xTiNbO5 enabled by redox active Ni exchange for potassium-ion batteries
Source: Chem Sci. 2025 Sep 15;16(41):19140–53. doi: 10.1039/d5sc04984a (PMC12445367; doi:10.1039/d5sc04984a)
Supplement: SC-016-D5SC04984A-s001 [file SC-016-D5SC04984A-s001.pdf]

Supplementary Information (SI) for Chemical Science.
This journal is © The Royal Society of Chemistry 2025

Supplementary Information

Enhanced ion intercalation in $Ni_xK_{1-2x}TiNbO_5$ enabled by redox active Ni exchange for potassium-ion batteries

Charlie A. F. Nason[a], Ajay Piriya Vijaya Kumar Saroja[b], Wanjun Ren[a], Yingkangzi Mei[a], Asma Sarguroh[a], Yupei Han[a], Yi Lu[a], Jamie Gould[a], Tim I. Hyde[a], Veronica Celorrio[c], Gopinathan Sankar[a], Yang Xu[a,]*

[a] Department of Chemistry, University College London, 20 Gordon Street, London WC1H 0AJ, UK

[b] Department of Chemistry, The University of Southampton, SO9 5NH, UK

[c] Diamond Light Source, Harwell Science and Innovation Campus, Didcot OX11 0DE, U.K.

E-mail: y.xu.1@ucl.ac.uk

Table S1. Lattice parameters and reliability factors (R_{wp} and *Goodness of Fit (GOF))* obtained from Rietveld refinements and Le Bail fits.

Sample	*Lattice parameters*			Reliability factors	
	a(Å)	b(Å)	c(Å)	R_{wp}	GOF
$KTiNbO_5$	6.43999(19)	3.79081(9)	18.4024(6)	10.75	2.18
$Ni_{(0.15)}K_{(0.7)}TiNbO_5$ A	6.4515(3)	3.79391(14)	18.7025(12)	9.66	1.6
$Ni_{(0.15)}K_{(0.7)}TiNbO_5$ B	6.475(4)	3.804(4)	21.36(3)	-	-
$Ni_{(0.25)}K_{(0.5)}TiNbO_5$ I	6.4626(22)	3.7801(14)	21.780(4)	12.53	1.55
$Ni_{(0.25)}K_{(0.5)}TiNbO_5$ II	6.4291(22)	3.7743(7)	18.934(9)	12.48	1.49
$Ni_{(0.25)}K_{(0.5)}TiNbO_5$ III	6.5315(14)	3.8792(10)	17.962(5)	12.689	1.5
$Ni_{(0.25)}K_{(0.5)}TiNbO_5$ IV	6.9206(9)	4.5316(9)	16.822(4)	15.2	2.75

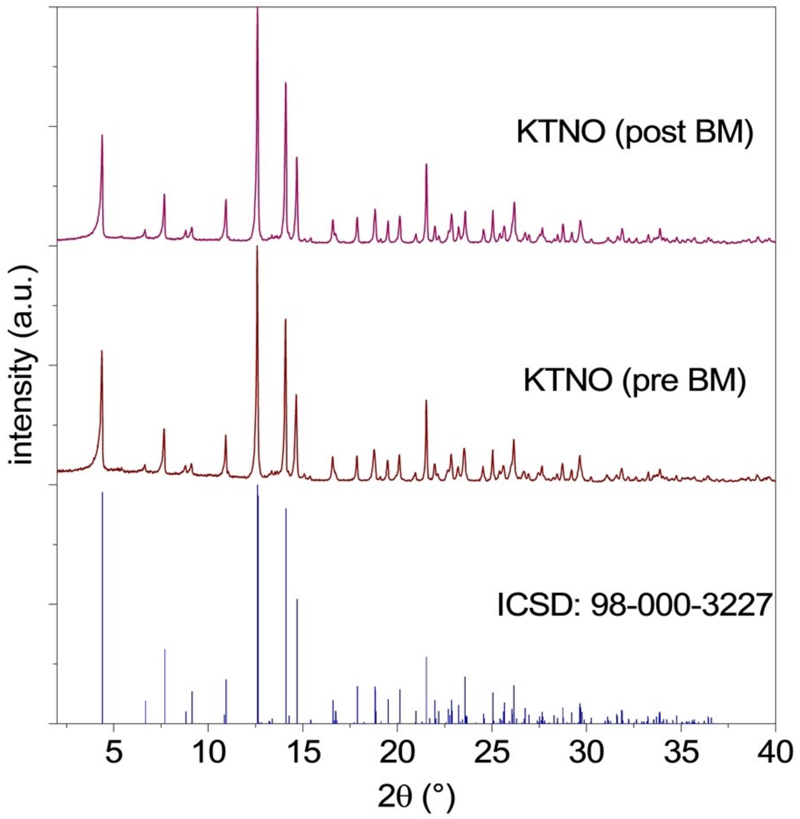

Figure S1. XRD patterns of KTNO pre and post ball milling (500 rpm, 5 mins running/2 mins rest x2, 1:1 volume ratio with IPA).

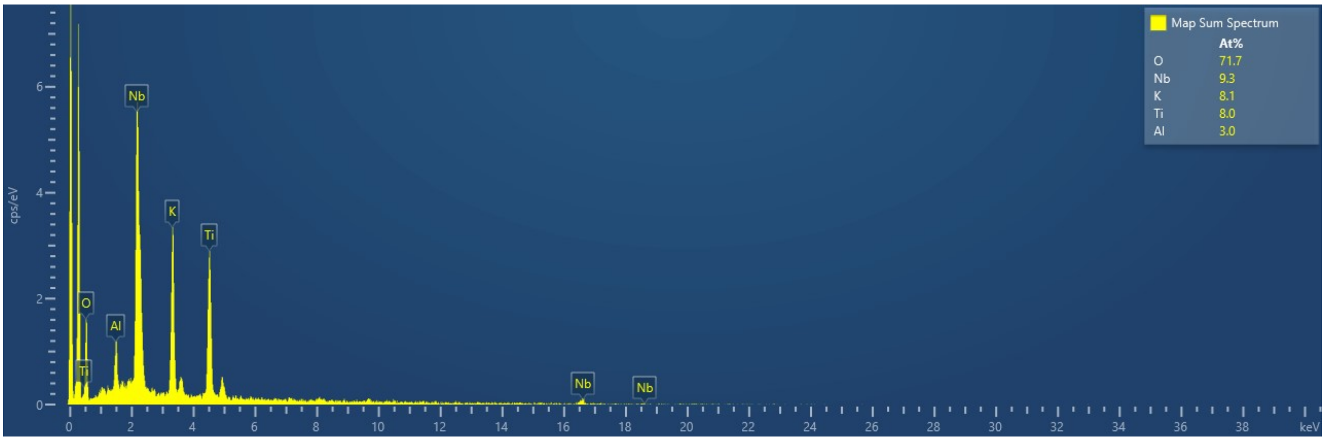

Figure S2. EDS spectrum of KTNO with the obtained At%.

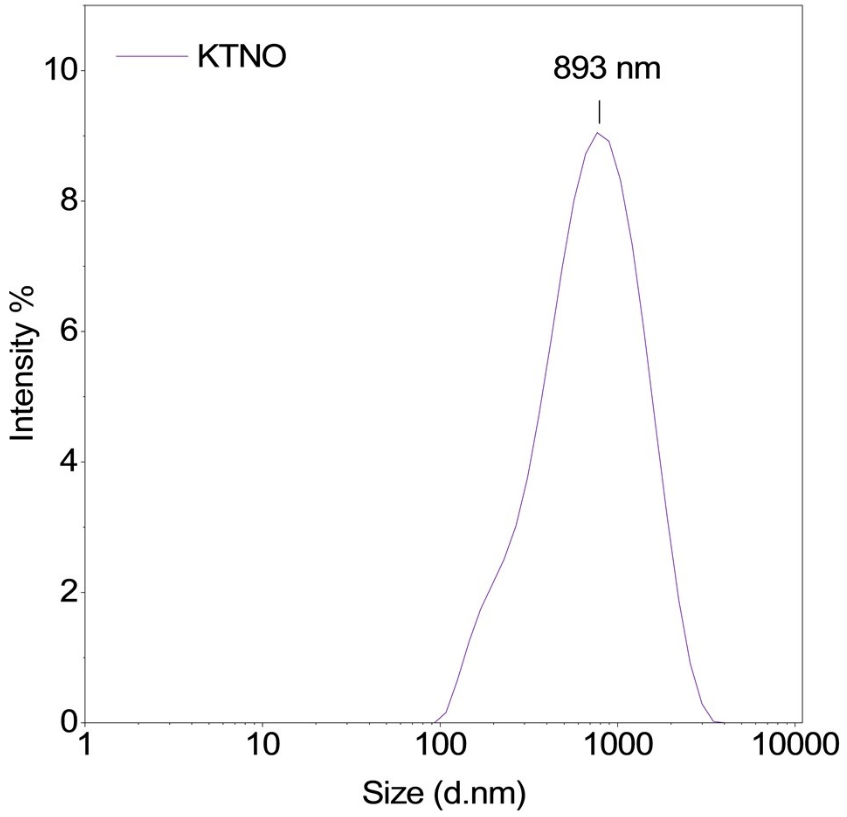

Figure S3. DLS spectrum of KTNO showing the particle size distribution in terms of % of number of particles at a particular size detected.

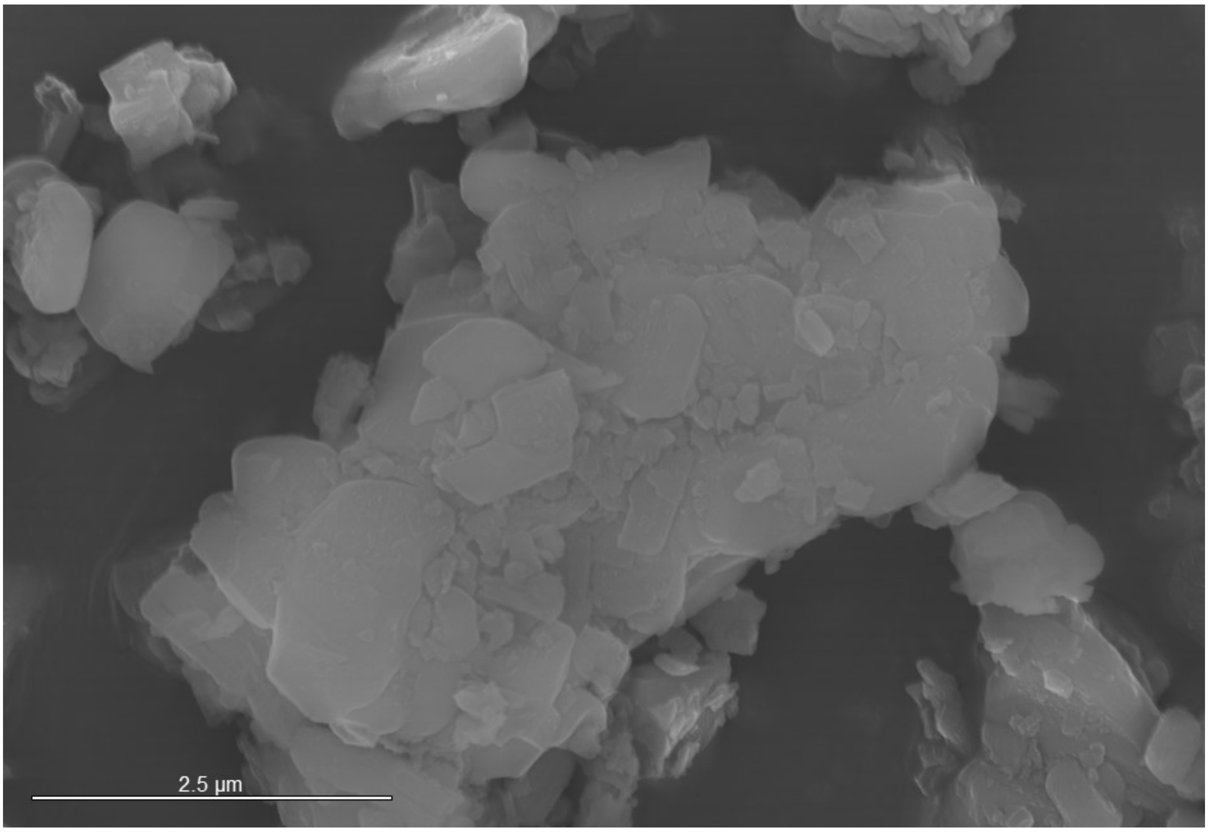

Figure S4. SEM image of Ni-KTNO-H, which matches the morphology of the post ball milled KTNO sample

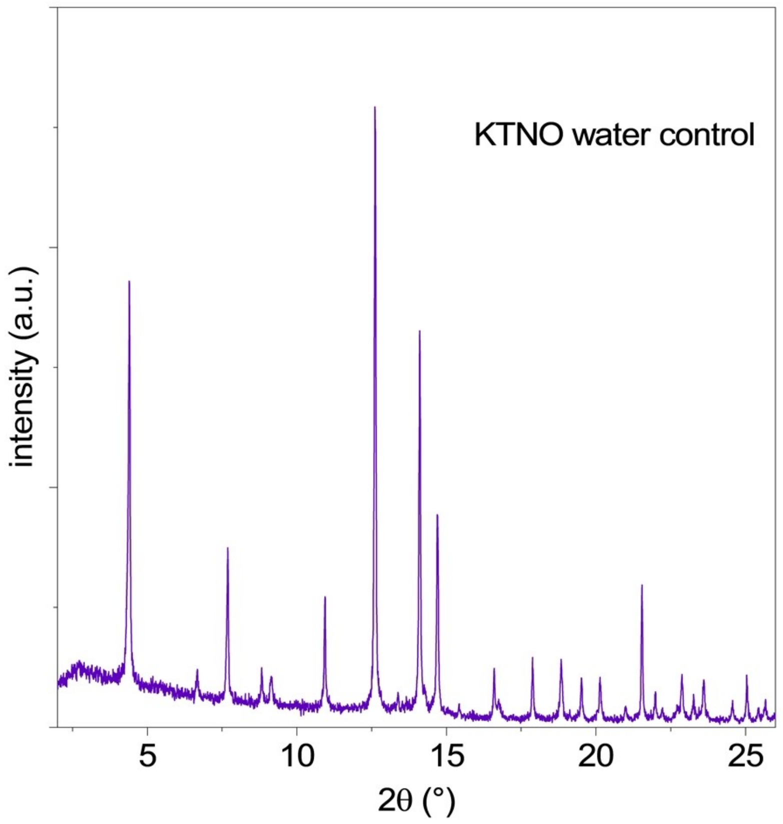

Figure S5. XRD pattern of KTNO control after stirring in water at 80 °C for 5 h, measured on Cu Kα1 radiation (1.54060 Å, 40 kV, 30 mA), then converted to Mo wavelength. Shows no peak pattern changes in the presence of water, ruling out water intercalation occurs without an exchange ion.

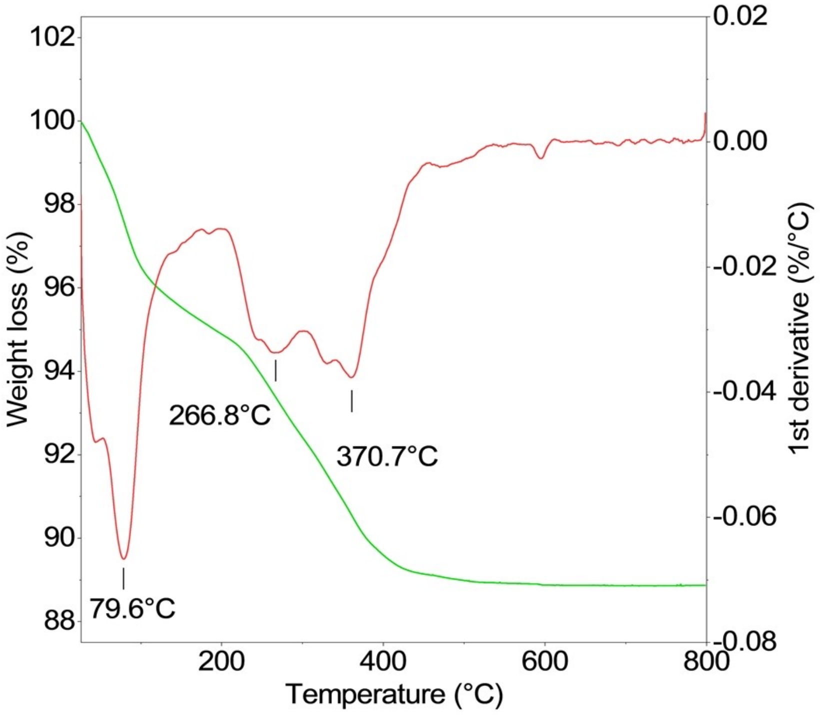

Figure S6. TGA spectrum of Ni-KTNO-H with plot of the first derivative, performed at 10° min^{-1} in air.

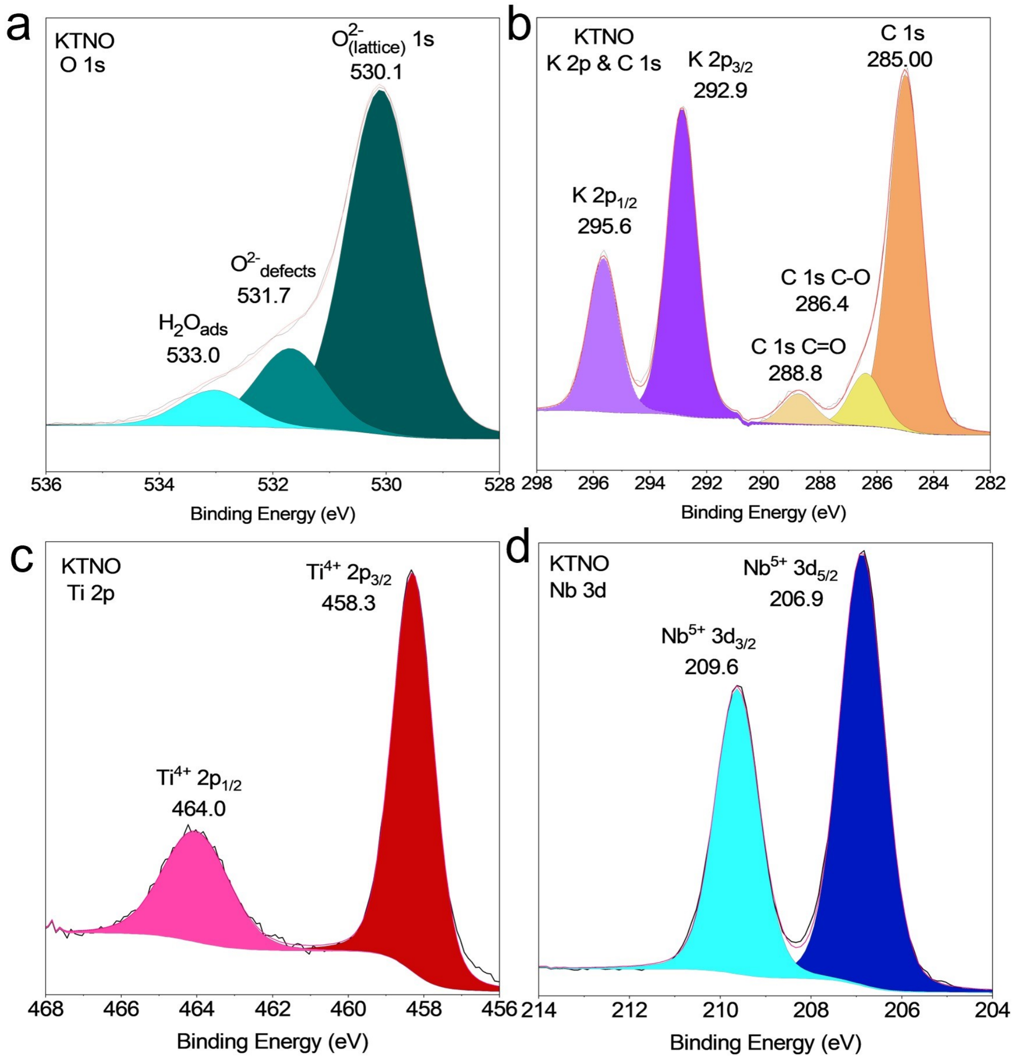

Figure S7. High resolution XPS spectra of a) O 1s, b) K 2p and C 1s, c) Ti 2p and d) Nb 3d of KTNO

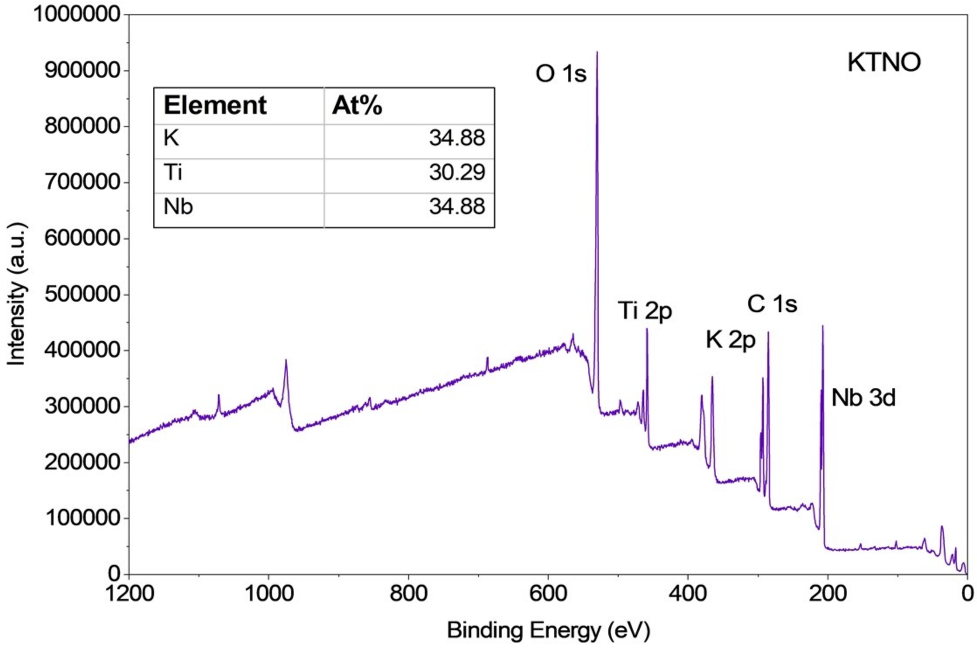

Figure S8. XPS survey spectrum of KTNO showing the surface elemental composition.

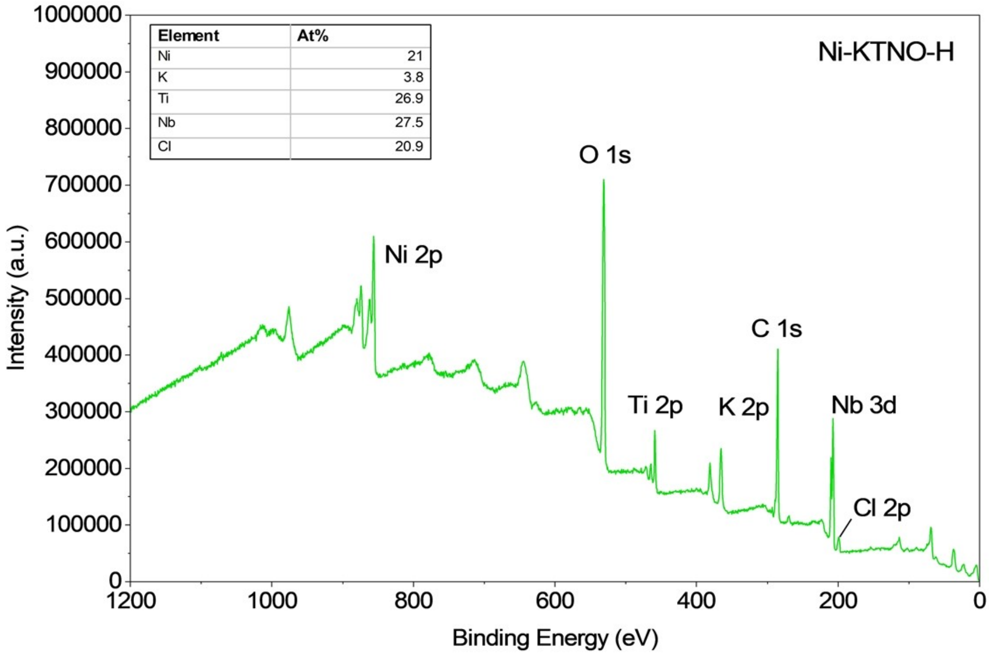

Figure S9. XPS survey spectrum of Ni-KTNO-H showing the surface elemental composition.

Table S2. Obtained EXAFS fit of χ(k) of the Ni K-edge.

Atom pair	N	R	σ^2	S_o^2
Ni-O	6.177	2.034	0.00810	0.891
Ni-K	2.961	3.210	0.00528	0.891
Ni-Ti	12.000	2.843	0.05641	0.891
Ni-Nb	12.000	3.126	0.04929	0.891

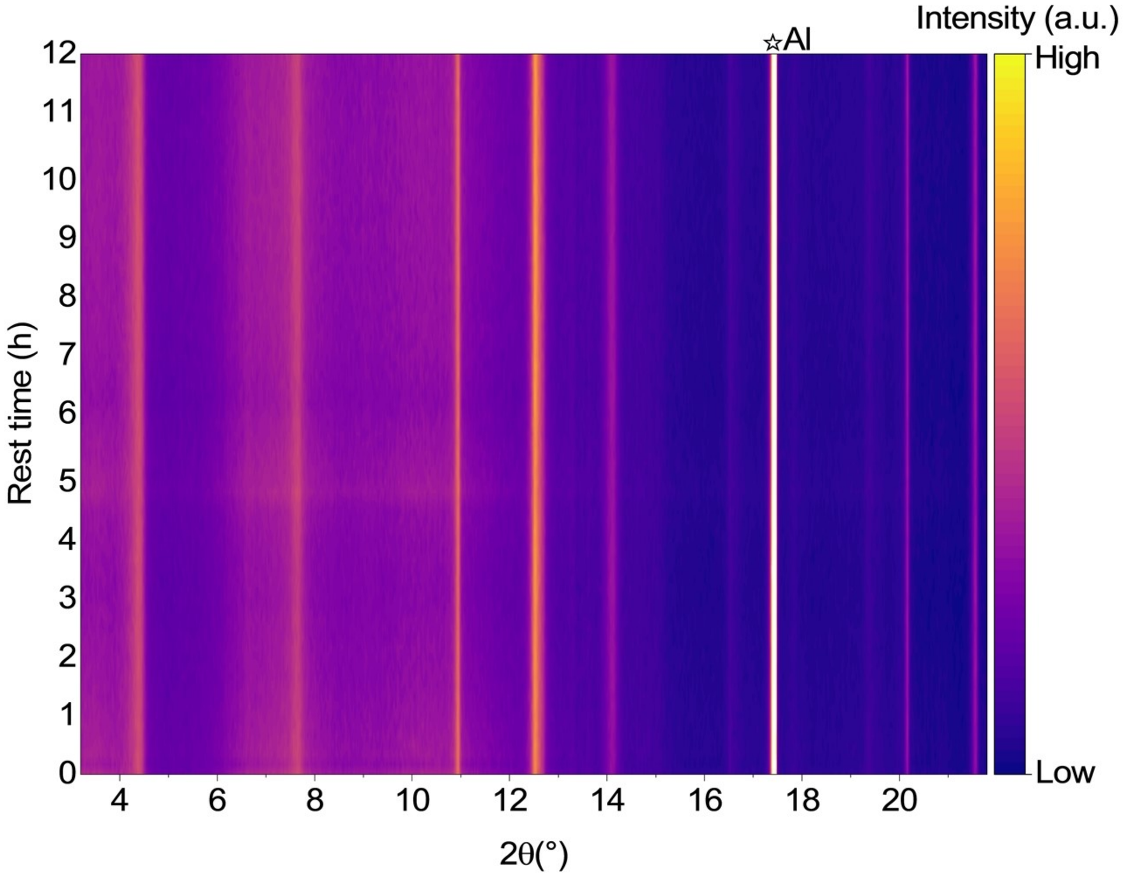

Figure S10. In-situ XRD of Ni-KTNO-H resting in electrolyte in an AMPIX cell for 12h, with the lack of any peak shifts or intensity changes indicating that the Ni-K exchange is irreversible under these conditions.

Table S3. EDS ratios of Ni-KTNO-H during the first cycle

Sample	Atomic %					Ratio	
	Ni	K	Ti	Nb	S	Ni:Ti	K:Ti
0.8 V Discharge	8.5	33.1	23.9	25.3	9.2	0.36	1.38
0.6 V Discharge	6.3	47.9	18.7	19	8.1	0.34	2.56
0.2 V Discharge	4.3	64.9	12.7	13.1	5.1	0.34	5.11
0.01 V Discharge	3.1	73.9	8.8	9.5	4.7	0.35	8.40
0.5 V Charge	2.8	75.3	8.2	9.5	4.2	0.34	9.18
1.8 V Charge	5.4	50.6	16.7	17.7	9.6	0.32	3.03
3 V Charge	6.2	43.3	18.7	19.4	12.4	0.33	2.32
X1000 cycles	3.1	80.3	9.4	7.2	-	0.33	8.54

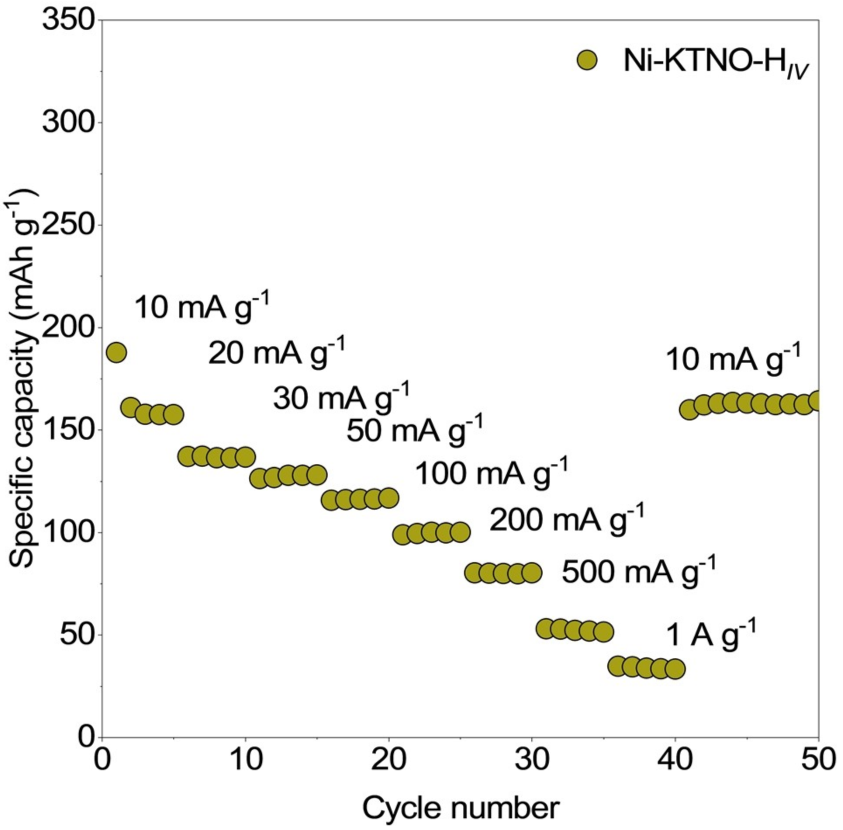

Figure S11. Rate performance of Ni-KTNO-H_{IV} from 10 mA g^{-1} to 1 A g^{-1}. The average capacity at the rates 10, 20, 30, 50, 100, 200, 500 and 1000 mA g^{-1} is 158, 136, 128, 116, 100, 80, 52, 34 mAh g^{-1}, respectively.

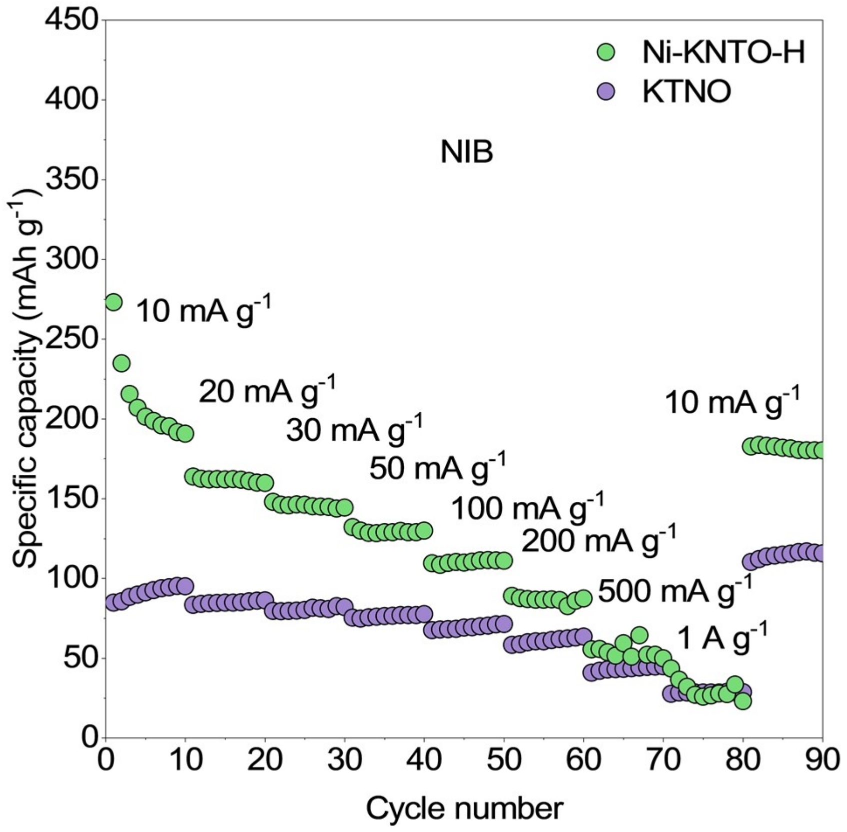

Figure S12. Rate performance of Ni-KTNO-H and KTNO in NIBs, from 10 mA g^{-1} to 1 A g^{-1}. The performance of Ni-KTNO-H at low rates exceeds pristine KTNO, but as the rate increases, the capacity of Ni-KTNO-H drops until it is equal to KTNO, which suggests the additional capacity is likely to generated from increased intercalation.

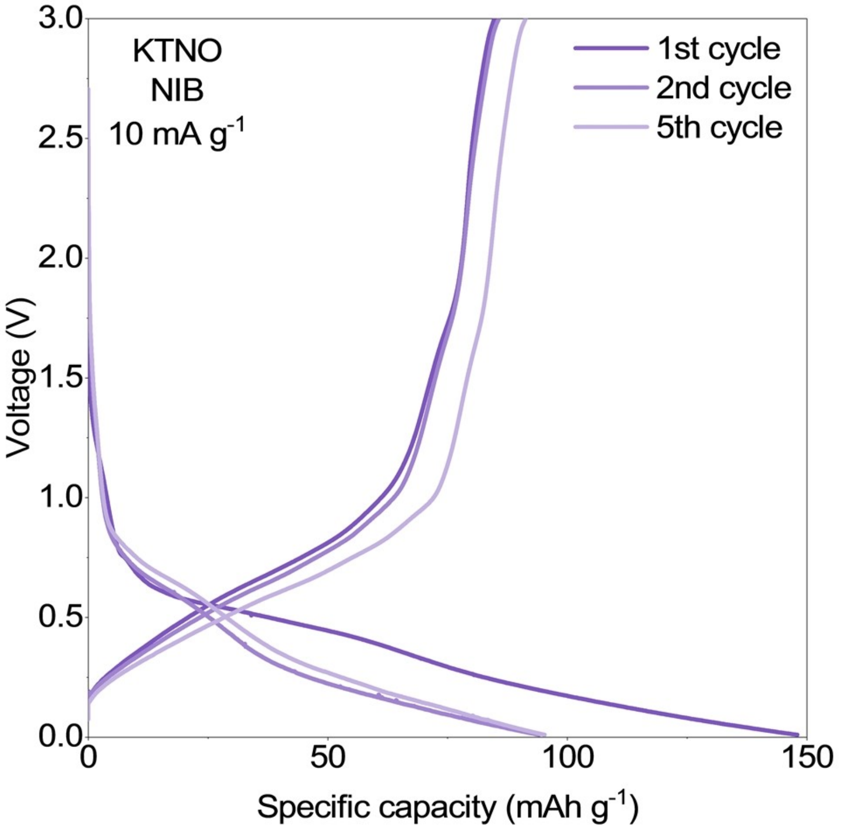

Figure S13. GCD curves of KTNO in NIBs, at 10 mA g^{-1}

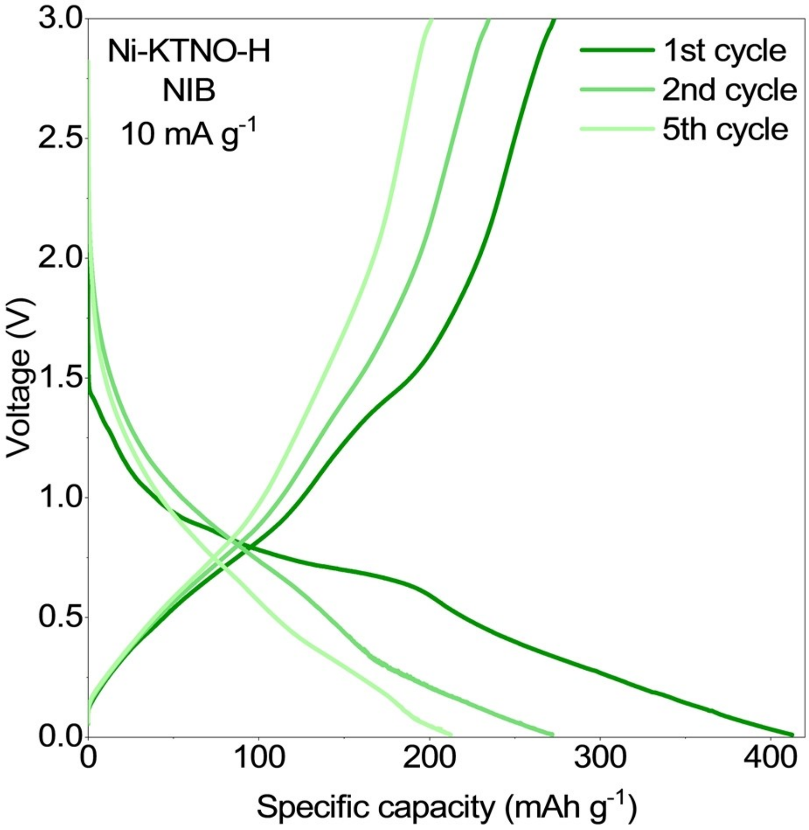

Figure S14. GCD curves of Ni-KTNO-H in NIBs, at 10 mA g^{-1}

Equation S1. $$i_p = av^b$$

Where i_p is the observed peak current, a is the proportionality constant, v is the scan rate and b is the exponent, with 0.5 indicating diffusion-controlled process and 1 capacitive-controlled process.

Equation S2. $$i(V) = k_1 v + k_2 v^{1/2}$$

Where $i(V)$ is the observed current at voltage V, k_1 is the capacitive coefficient, v is the scan rate and k_2 is the diffusion coefficient, giving the proportion of each to the observed current.

Equation S3. $$D = \frac{4}{9\pi} \cdot \left(\frac{E_4 - E_0}{E_2 - E_0}\right)^2 \cdot \frac{{r_p}^2}{t_p}$$

Where D is the diffusion coefficient, E_0 is the potential pre-pulse, E_2 is the potential directly after pulse ends, showing the IR drop and $E_4$is the equilibrium potential after rest. r_p corresponds to the radius of the particle (approximating as spherical) and t_p is the length of the pulse.

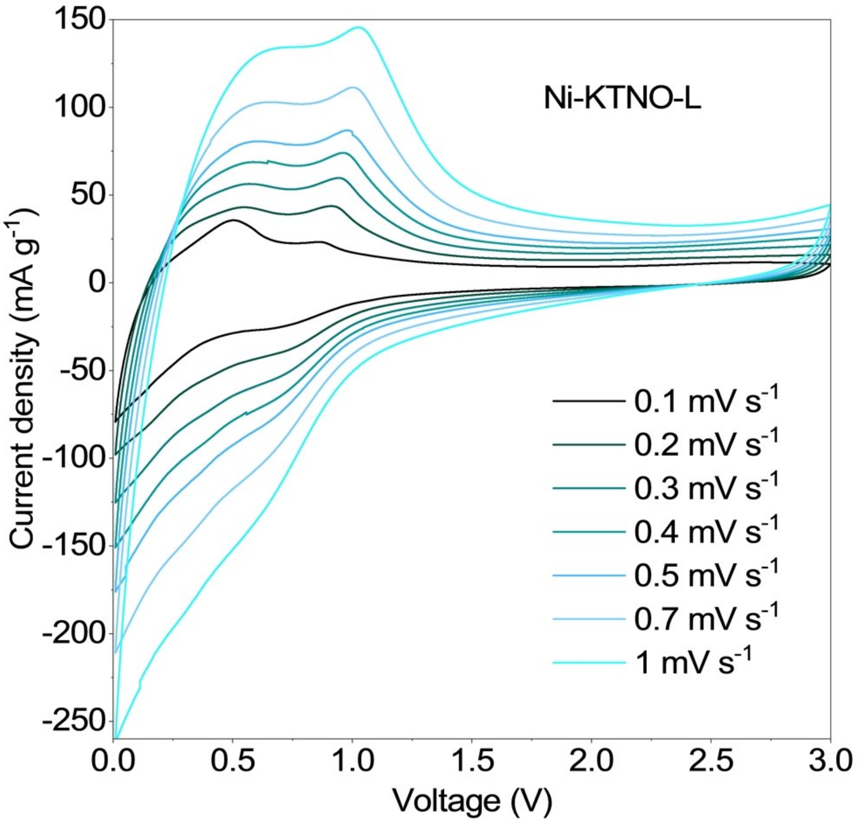

Figure S15. CV curves of Ni-KTNO-L at 0.1 mV s^{-1} to 1 mV s^{-1}

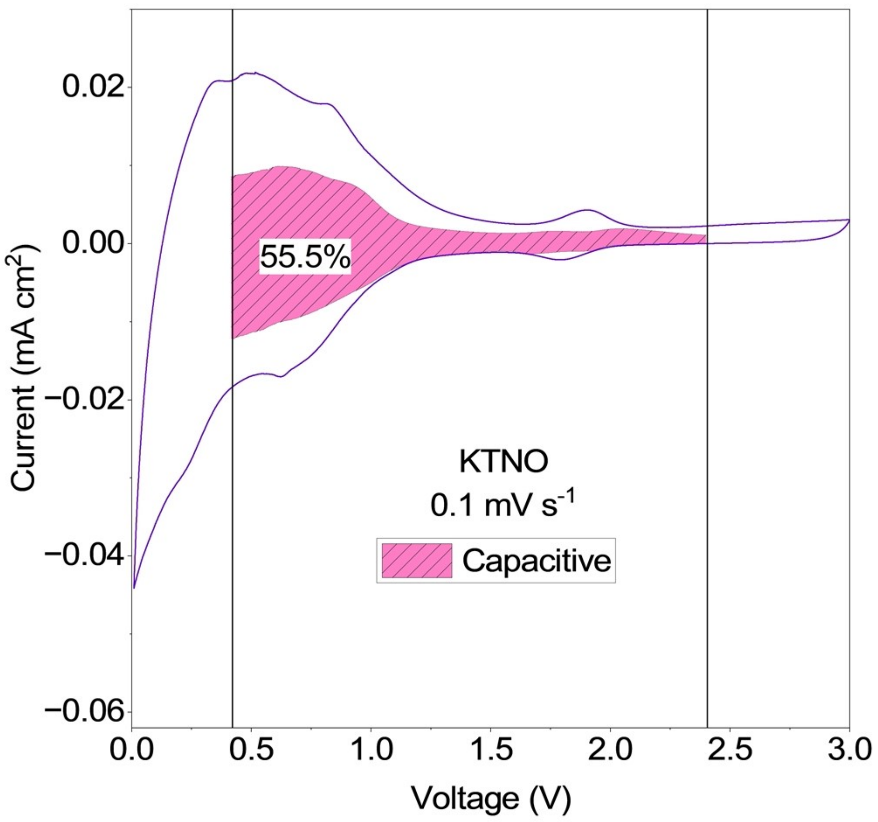

Figure S16. Capacitive contribution of KTNO at 0.1 mV s^{-1}

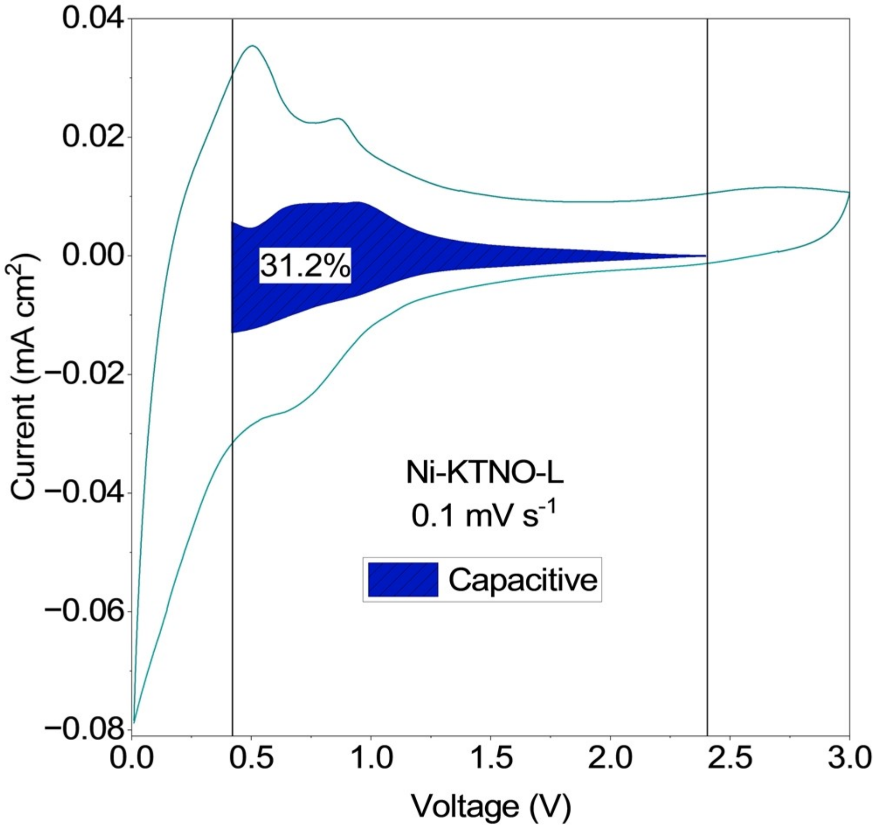

Figure S17. Capacitive contribution of Ni-KTNO-L at 0.1 mV s^{-1}

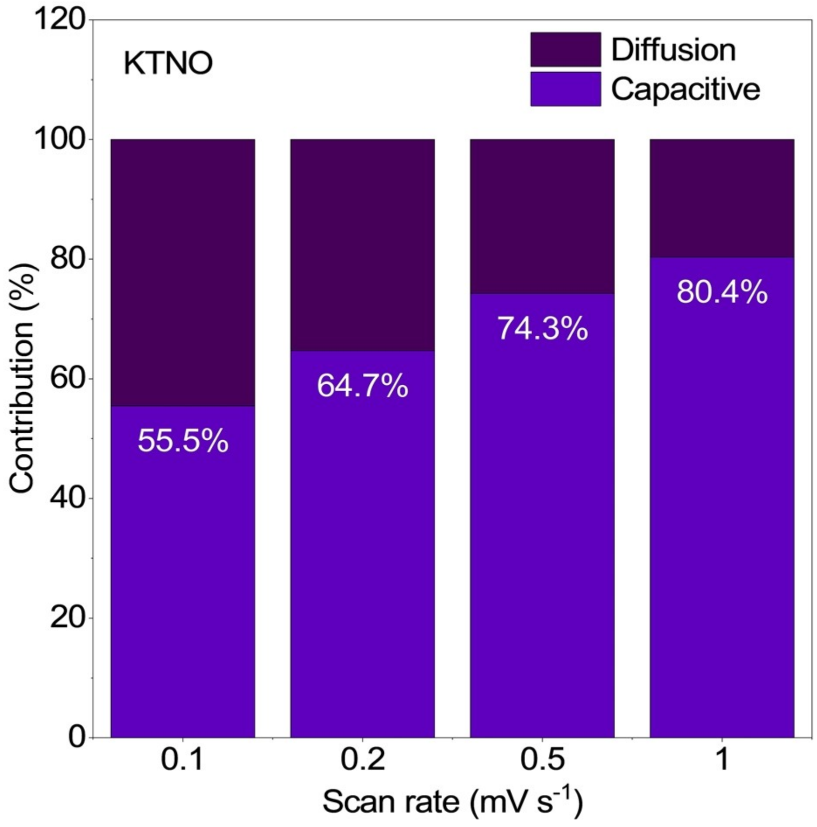

Figure S18. Capacitive contribution of KTNO at 0.1, 0.2, 0.5 and 1 mV s^{-1}

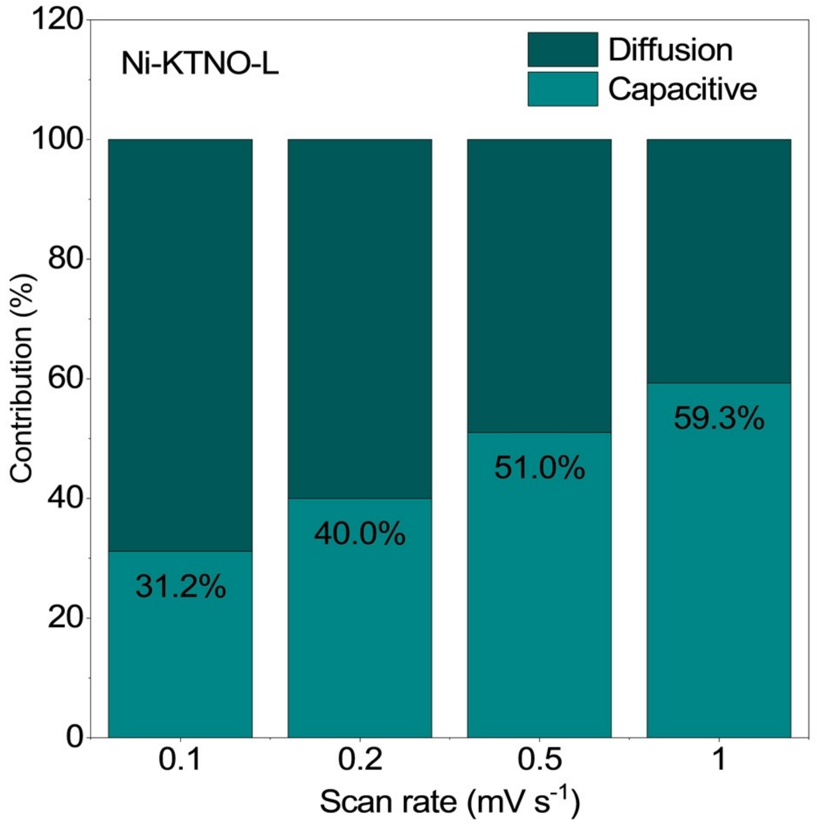

Figure S19. Capacitive contribution of Ni-KTNO-L at 0.1, 0.2, 0.5 and 1 mV s^{-1}

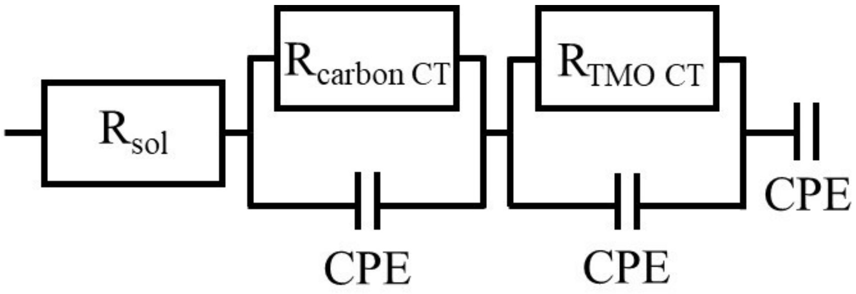

Figure S20. Equivalent circuit used for fitting EIS spectra. This includes the solution resistance (R_{sol}), the charge transfer resistance of the Super P and the transition metal oxide active material. A constant phase element is used to approximate the Warburg impedance, as pseudocapacitors exhibit non-ideal diffusion behaviour.

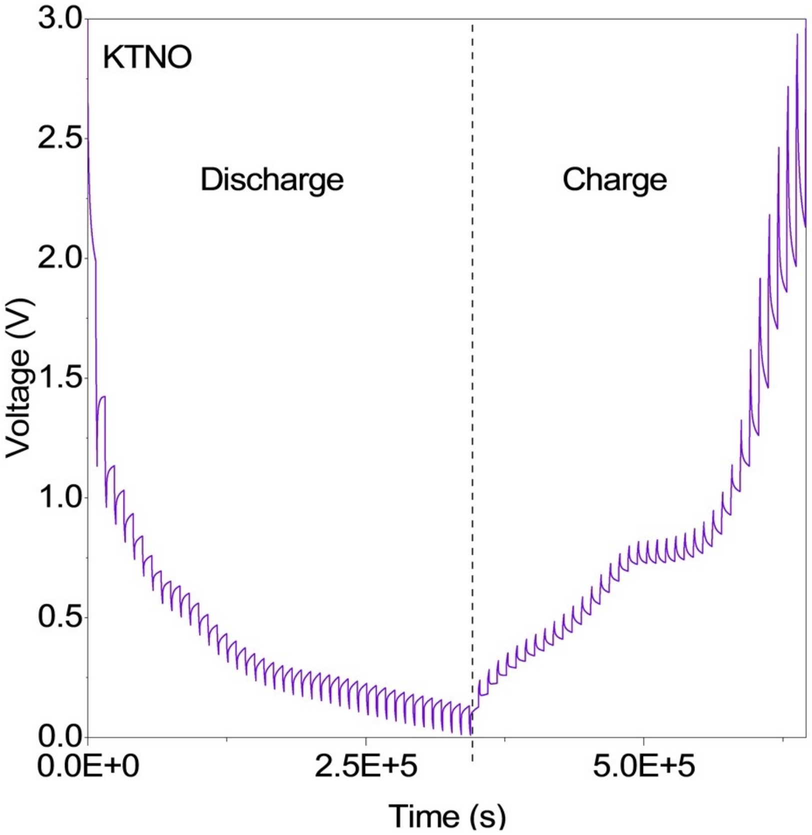

Figure S21. GITT curves of KTNO, with a 10 mA g^{-1} pulse with a duration of 20 mins.

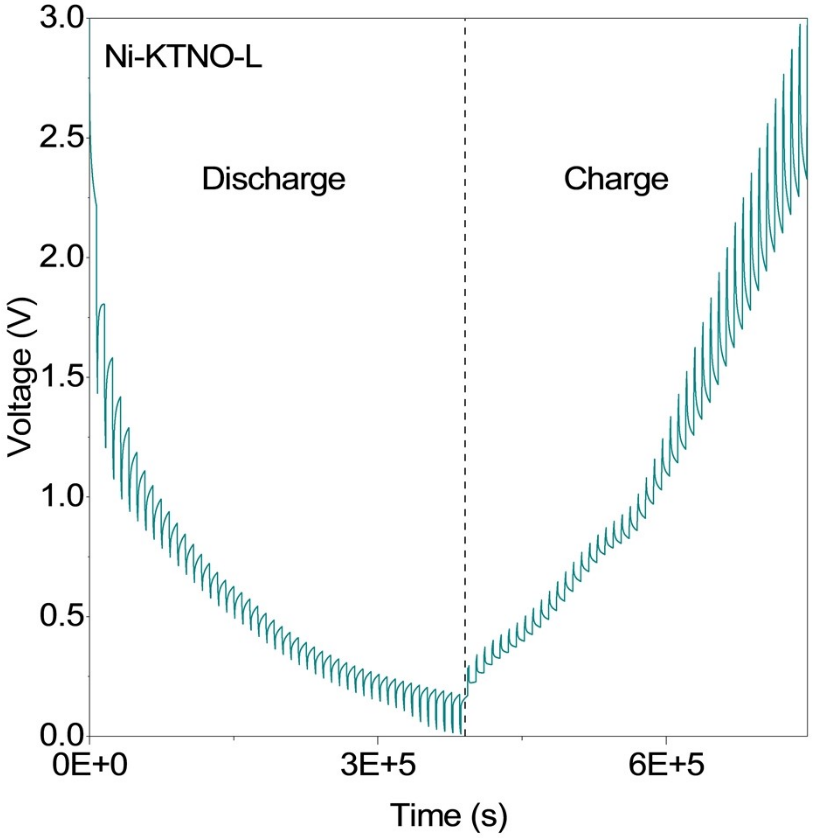

Figure S22. GITT curves of Ni-KTNO-L, with a 10 mA g^{-1} pulse with a duration of 20 mins.

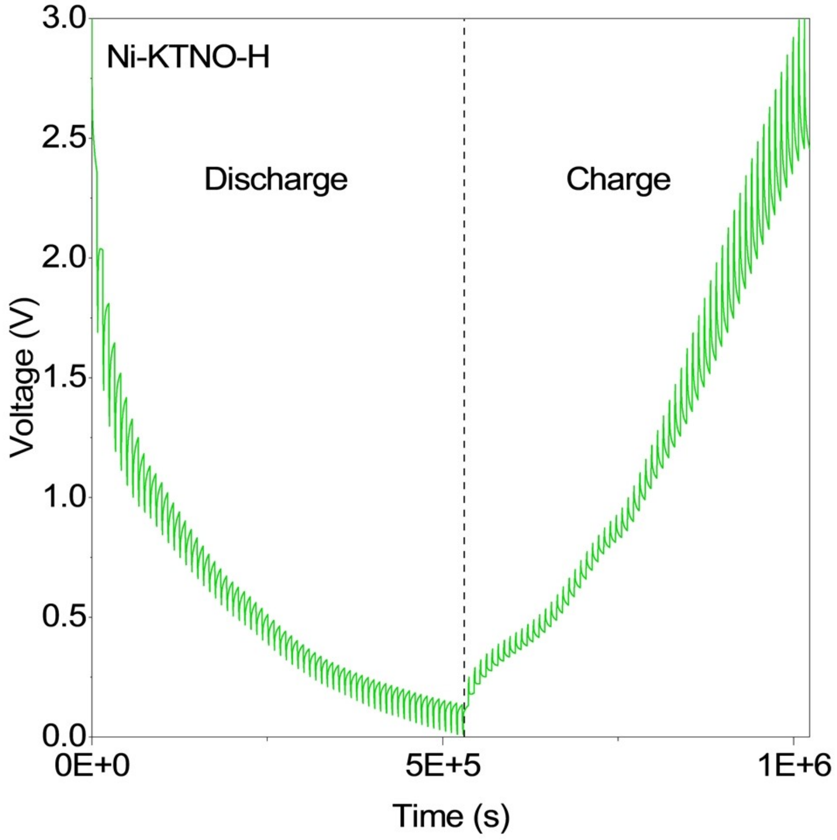

Figure S23. GITT curves of Ni-KTNO-H, with a 10 mA g^{-1} pulse with a duration of 20 mins.

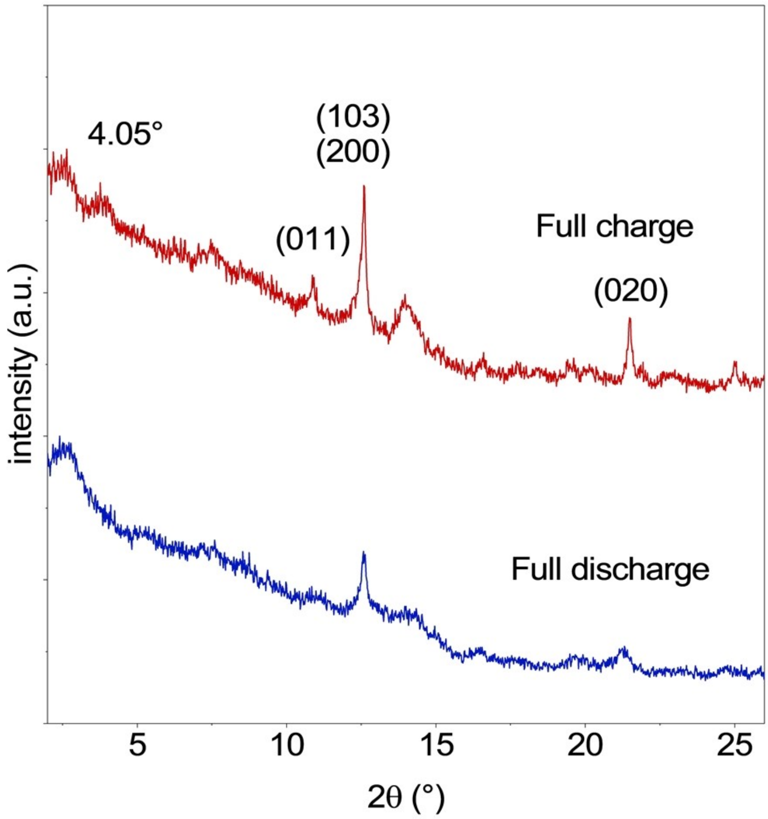

Figure S24. Ex-situ XRD of Ni-KTNO-H taken at full discharge (0.01 V) and full charge (3 V). These show the loss of the (002) peak at full discharge, along with broadening of all peaks. At full charge, the (002) is observed to reappear, albeit very broad and ill-defined, along with the (011) and the (020) reforming.

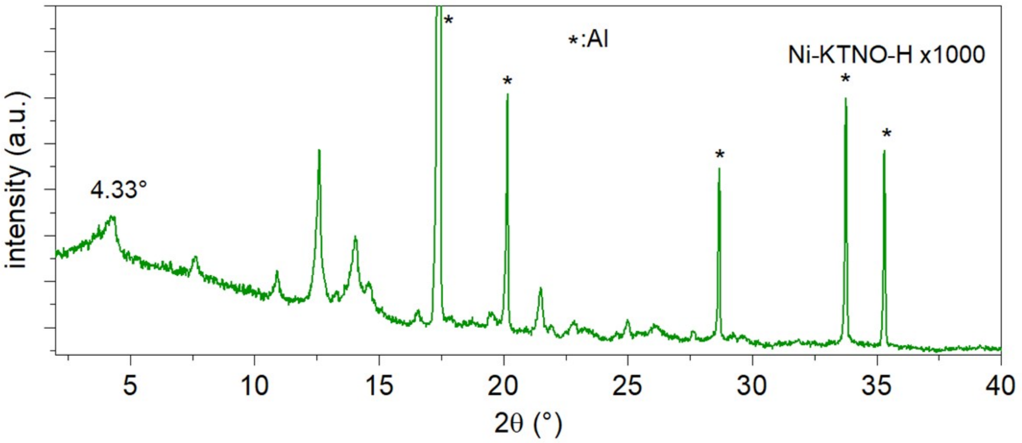

Figure S25. Ex-situ XRD of Ni-KTNO-H after 1000 cycles at 100 mA g^{-1}.

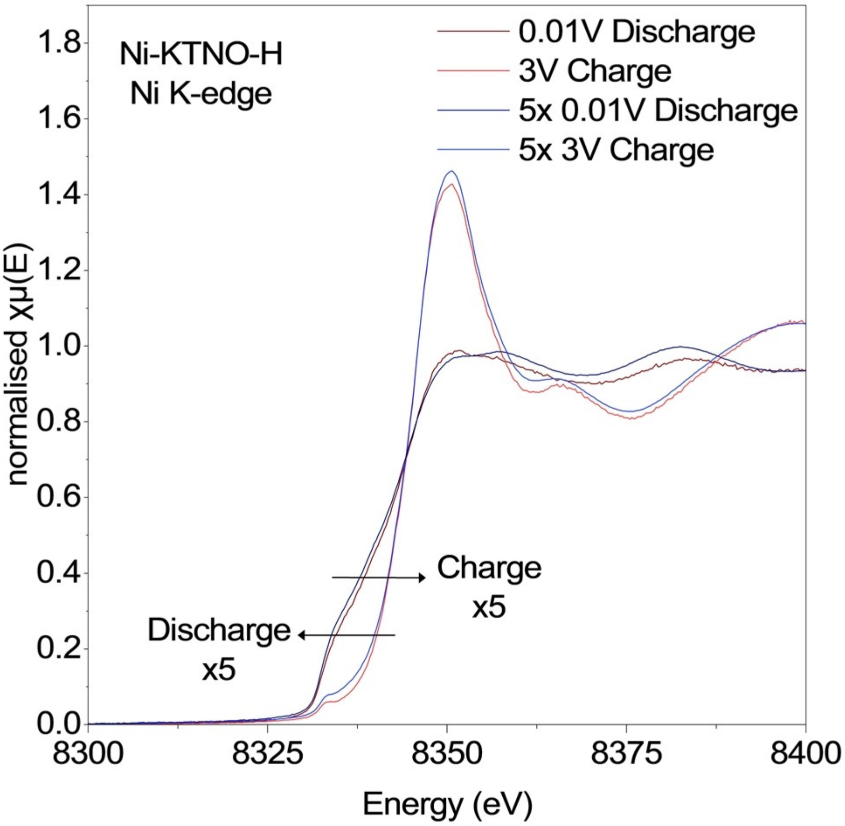

Figure S26. Ni K-edge XANES spectra of Ni-KTNO-H at full discharge, full charge and after 5 cycles.

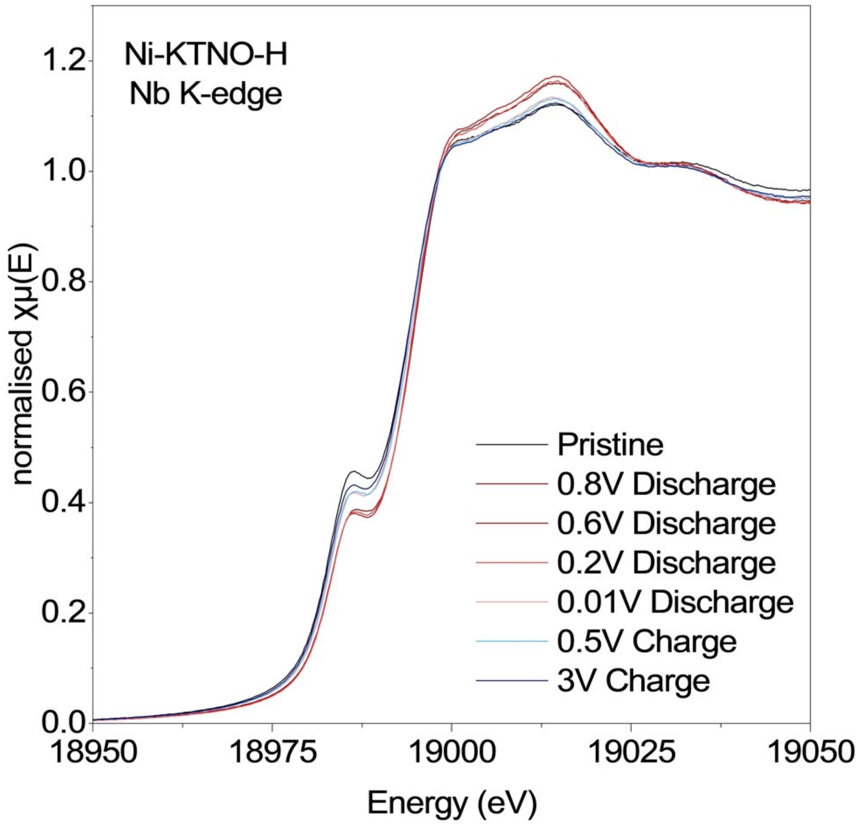

Figure S27. Nb K-edge XANES spectra of Ni-KTNO-H during the charge-discharge process

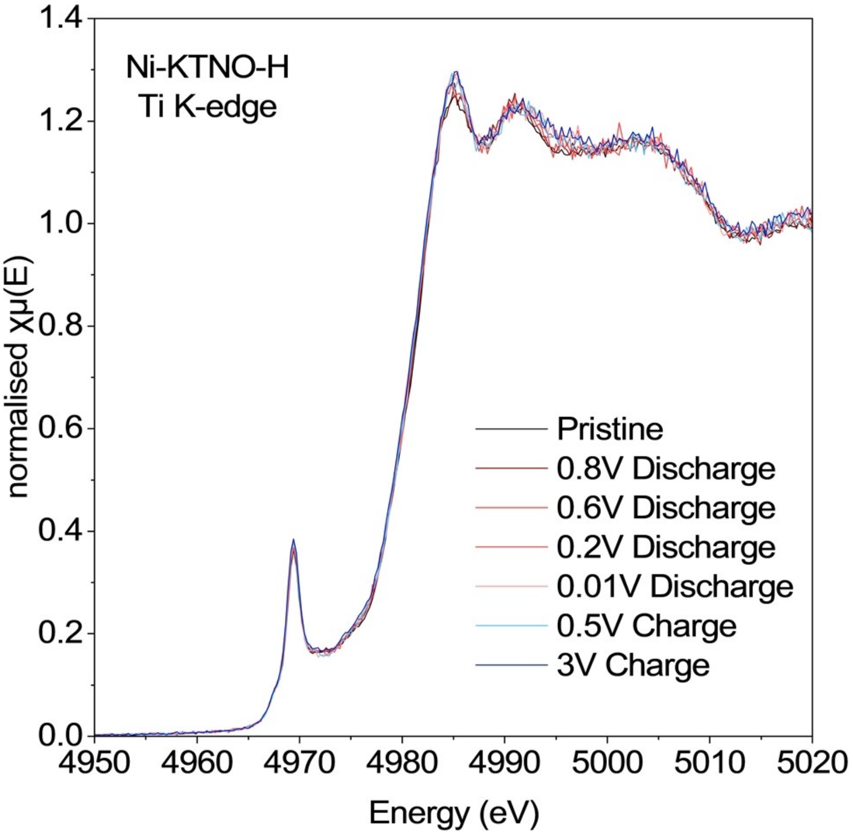

Figure S28. Ti K-edge XANES spectra of Ni-KTNO-H during the charge-discharge process
